# Diagnostic Accuracy of Kato-Katz and FLOTAC for Assessing Anthelmintic Drug Efficacy

**DOI:** 10.1371/journal.pntd.0001036

**Published:** 2011-04-12

**Authors:** Stefanie Knopp, Benjamin Speich, Jan Hattendorf, Laura Rinaldi, Khalfan A. Mohammed, I. Simba Khamis, Alisa S. Mohammed, Marco Albonico, David Rollinson, Hanspeter Marti, Giuseppe Cringoli, Jürg Utzinger

**Affiliations:** 1 Department of Epidemiology and Public Health, Swiss Tropical and Public Health Institute, Basel, Switzerland; 2 University of Basel, Basel, Switzerland; 3 Department of Pathology and Animal Health, Regional Center for Monitoring Parasites (CREMOPAR), Regione Campania, University of Naples “Federico II”, Naples, Italy; 4 Helminth Control Laboratory Unguja, Ministry of Health and Social Welfare, Zanzibar, United Republic of Tanzania; 5 Ivo de Carneri Foundation, Milano, Italy; 6 Public Health Laboratory Ivo de Carneri, Chake-Chake, Pemba, United Republic of Tanzania; 7 Wolfson Wellcome Biomedical Laboratories, Biomedical Parasitology Division, Department of Zoology, Natural History Museum, London, United Kingdom; 8 Department of Medical Services and Diagnostic, Swiss Tropical and Public Health Institute, Basel, Switzerland; University of Oklahoma Health Sciences Center, United States of America

## Abstract

**Background:**

Sensitive diagnostic tools are required for an accurate assessment of prevalence and intensity of helminth infections in areas undergoing regular deworming, and for monitoring anthelmintic drug efficacy. We compared the diagnostic accuracy of the Kato-Katz and FLOTAC techniques in the frame of a drug efficacy trial.

**Methodology/Principal Findings:**

Stool samples from 343 Zanzibari children were subjected to duplicate Kato-Katz thick smears and the FLOTAC basic technique in a baseline screening in early 2009. The FLOTAC showed a higher sensitivity than the Kato-Katz method for the diagnosis of *Trichuris trichiura* (95% *vs.* 88%, p = 0.012) and *Ascaris lumbricoides* (88% *vs.* 68%, p = 0.098), but a lower sensitivity for hookworm diagnosis (54% *vs.* 81%, p = 0.006). Considering the combined results from both methods as ‘gold’ standard, the prevalences of *T. trichiura*, hookworm and *A. lumbricoides* were 71% (95% confidence interval (CI): 66–75%), 22% (95% CI: 17–26%) and 12% (95% CI: 8–15%), respectively. At follow-up, 3–5 weeks after 174 among the 269 re-examined children were administered anthelmintic drugs, we observed cure rates (CRs) against *A. lumbricoides*, hookworm and *T. trichiura* of 91% (95% CI: 80–100%), 61% (95% CI: 48–75%) and 41% (95% CI: 34–49%), respectively, when using the Kato-Katz method. FLOTAC revealed lower CRs against *A. lumbricoides* (83%, 95% CI: 67–98%) and *T. trichiura* (36%, 95% CI: 29–43%), but a higher CR against hookworm (69%, 95% CI: 57–82%). These differences, however, lacked statistical significance. Considerable differences were observed in the geometric mean fecal egg counts between the two methods with lower egg reduction rates (ERRs) determined by FLOTAC.

**Conclusion/Significance:**

Our results suggest that the FLOTAC technique, following further optimization, might become a viable alternative to the Kato-Katz method for anthelmintic drug efficacy studies and for monitoring and evaluation of deworming programs. The lower CRs and ERRs determined by FLOTAC warrant consideration and could strategically impact future helminth control programs.

## Author Summary

In areas where parasitic worm infections have been successfully reduced as a result of deworming campaigns, the level of infections and drug efficacy must be carefully monitored. For this purpose, diagnostic methods with a high sensitivity are needed. We compared the accuracy of the widely used Kato-Katz method with the more recently developed FLOTAC technique for the diagnosis of parasitic worms. Our study was done with children on Zanzibar island, Tanzania, within the frame of an anthelmintic drug efficacy study. We collected stool samples from 343 children in two primary schools before and after treatment and examined the stool samples with both methods. FLOTAC showed a higher sensitivity than Kato-Katz for the diagnosis of roundworm and whipworm, but a lower sensitivity for hookworm diagnosis. The cure rates determined by FLOTAC were lower for roundworm and whipworm when compared with Kato-Katz. The opposite was found for hookworm. Egg reduction rates were generally lower when the FLOTAC technique was used. Our results suggest that the FLOTAC method, after additional optimization, can become a viable alternative to the Kato-Katz method for anthelmintic drug efficacy studies and for monitoring and evaluation of deworming programs.

## Introduction

Current estimates suggest that soil-transmitted helminths might still affect over a quarter of the world's population and inflict a huge public health burden, particularly on rural and deprived urban communities in the developing world [1]–[3]. Efforts are underway to reduce morbidity due to soil-transmitted helminthiasis, with an ambitious target to administer anthelmintic drugs regularly to at least 75% and up to 100% of school-aged children and other high-risk groups [4]. One region where this goal was already met in 2006 is Zanzibar, a group of islands forming part of the United Republic of Tanzania [5]. Indeed, helminth control programs have been implemented in Zanzibar for 20 years and have resulted in marked decreases in prevalence, intensity and morbidity due to soil-transmitted helminthiasis, urinary schistosomiasis, and lymphatic filariasis [6]–[9].

The diagnosis of soil-transmitted helminth infections with direct parasitological methods based on egg detection in stool is unreliable among infected individuals who harbor only a few intestinal worms, since egg output is much lower than among heavily infected individuals. Hence, in settings where helminth control programs have been implemented and infection intensities dropped as a result of regular deworming, diagnostic methods with a high sensitivity are needed for an accurate assessment of the actual prevalence and intensity of soil-transmitted helminth infections [10]. Sensitive diagnostic tools are also mandatory for monitoring drug efficacy and to detect the emergence and spread of drug resistance [11], [12]. The Kato-Katz method [13] is a widely used diagnostic tool, which provides estimates of population prevalence and infection intensity, and facilitates anthelmintic drug efficacy assessment in clinical trials and monitoring and evaluation of community-based control programs. However, due to the small amount of stool examined (i.e., 41.7 mg), the Kato-Katz method shows a low sensitivity when infection intensities are light, which is common after deworming [14]. The sensitivity can be improved by examining multiple Kato-Katz thick smears produced from a single stool sample or by examining multiple stool samples [15]–[17].

With the recently developed FLOTAC method, up to 1 g of stool can be examined – 24 times more than with a single Kato-Katz thick smear – and standard protocols are now available [18]. The FLOTAC technique showed a higher sensitivity than multiple Kato-Katz thick smears for the diagnosis of soil-transmitted helminth infections in previous studies [19]–[22]. Moreover, FLOTAC outperformed the McMaster technique qualitatively and quantitatively for hookworm diagnosis [23]. What is not known, though, is how the FLOTAC technique performs within anthelmintic drug efficacy trials, and whether it might be utilized for monitoring and evaluation of helminth control programs that are shifting the tactic from morbidity control to infection and transmission control, and eventually local elimination.

The objective of this study was to compare the accuracy of the Kato-Katz method with the FLOTAC basic technique for the detection of soil-transmitted helminth infections within a randomized controlled trial on anthelmintic drug efficacy and safety carried out in Zanzibar in early 2009 [24]. The diagnostic accuracy of each method was determined before and after treatment and anthelmintic drug efficacy was estimated according to the method used.

## Methods

### Ethics statement

The study presented here was embedded in a randomized controlled trial with the protocol being reviewed by the institutional research commission of the Swiss Tropical and Public Health Institute (Basel, Switzerland). Ethical approval was given by the ethics committee of Basel (EKBB, reference no. 13/09) and the Ministry of Health and Social Welfare of Zanzibar (MoHSW, reference no. ZAMEC/0001/09). The study is registered at controlled-trials.com, identifier ISRCTN08336605.

The directors and teachers of the primary schools in Kinyasini and Kilombero were informed about the purpose and procedures of the study. The study was explained in lay terms to the school children in their local language (Kiswahili). An informed consent sheet, including study information and the fact that participation was voluntary, was distributed to each child. Written informed consent was obtained from parents or guardians prior to stool sampling. Additionally, oral assent was sought from children.

At the end of the study in May 2009, all children attending the two schools were offered free anthelmintic drugs, as part of the regular deworming done by the Helminth Control Laboratory Unguja (HCLU). Single oral doses of albendazole (400 mg) and praziquantel (40 mg/kg) were administered to school-aged children for preventing morbidity due to soil-transmitted helminthiasis and urinary schistosomiasis, respectively.

### Study area and population

The study was carried out on Unguja island, Zanzibar, in the first half of 2009 within the frame of a randomized controlled trial to assess the efficacy and safety of different anthelmintic drugs against *Trichuris trichiura* and other soil-transmitted helminth infections [24]. For the assessment of anthelmintic drug efficacy according to the diagnostic method used, we aimed at enrolling at least 200 *T. trichiura*-positive individuals [25]. In view of the sample size calculations for our clinical trial, we aimed at screening 2000 children to identify 600 *T. trichiura*-infected subjects, assuming a prevalence of 30% [8]. All children attending the primary schools of Kilombero and Kinyasini in district North A, located 30–40 km from Zanzibar Town, were eligible to submit stool samples. To reach the number of 200 *T. trichiura*-infected children for assessing anthelmintic drug efficacy, not only with the Kato-Katz, but also with the FLOTAC technique, we systematically preserved ∼1 g of every third stool sample collected at the baseline screening for subsequent FLOTAC examinations. Since the *T. trichiura* prevalence turned out to be >50%, we stopped recruiting children during our baseline survey after we had included 1240 children in the study. Among these children, 1066 (86%) had submitted one stool sample; 750 from Kinyasini and 316 from Kilombero.

### Field and laboratory procedures

The baseline screening was carried out as follows: in early March 2009, over a period of 3 weeks, every morning between 08:00 and 09:30 hours, ∼120 children were called from class and given a stool collection container labeled with unique identification numbers (IDs). Children were asked to return the container filled with a lime-sized own fresh morning stool sample the following day. Upon collection, filled stool containers were ordered by increasing IDs in a wooden transport-shelf and promptly transferred to the HCLU in Zanzibar Town.

At HCLU, duplicate Kato-Katz thick smears were prepared from each stool sample, using standard 41.7 mg templates [13]. All Kato-Katz thick smears were examined quantitatively by one of four experienced laboratory technicians for hookworm eggs after a clearing time of 20–40 min in the morning and by another one of four experienced laboratory technicians for *T. trichiura* and *Ascaris lumbricoides* eggs in the afternoon. Slides were numbered with the child's ID plus letter A or B, and each microscopist adhered to either the A or B series to avoid duplicate reading of the same stool sample by the same technician.

With regard to the FLOTAC method, ∼1 g of stool, systematically obtained from each third stool sample in the transport-shelf, was weighed in a plastic tube, filled with 10 ml of 5% formaldehyde. Stool samples were suspended with a wooden spatula and stored at room temperature until further use.

Three to 9 weeks after the collection of the first stool sample, children who had their stool samples examined both by the Kato-Katz and FLOTAC techniques were invited to submit a second stool sample. Samples were again processed with duplicate Kato-Katz, and ∼1 g of stool was preserved in 5% formaldehyde and stored at room temperature. Of note, children with a *T. trichiura* infection as identified by duplicate Kato-Katz thick smear readings at baseline and meeting other inclusion criteria, were treated with one of the following four drug regimens: (i) albendazole (400 mg) plus placebo; (ii) albendazole plus ivermectin (200 µg/kg); (iii) mebendazole (500 mg) plus placebo; and (iv) mebendazole plus ivermectin. Drugs were administered shortly after the end of the baseline screening in late March 2009. Follow-up stool samples from children who had received anthelmintic drugs were collected within 3–5 weeks after treatment.

The administration of albendazole and praziquantel to all school children in Kinyasini and Kilombero (and other schools) regardless of whether or not children participated in our study was carried out by members of the HCLU in late May 2009, when all available follow-up stool samples had been collected.

After completion of the trial in late May 2009, in the last 2 weeks of the study, all formaldehyde-preserved stool samples were examined with the FLOTAC basic technique [18]. Since FLOTAC had not been implemented at HCLU before, all 22 laboratory workers, including eight microscopists, underwent a 3-day training workshop with two FLOTAC specialists from Italy to become acquainted with this new diagnostic procedure.

We used flotation solution no. 4 (FS4; sodium nitrate: NaNO_3_ 315 g plus 685 ml H_2_O; specific gravity (s.g.) = 1.20) in light of our preceding results for the diagnosis of soil-transmitted helminth infections [18], [19], [21]. Each preserved stool suspension was pressed through a tea sieve using a wooden spatula and adding 10 ml of 0.9% NaCl. The supernatant was equally distributed in two labeled 15 ml plastic tubes and centrifuged for 3 min at 170 *g* in a Hettich EBA centrifuge (Tuttlingen, Germany). Subsequently, the supernatant was discarded and each tube filled to the 6 ml mark with FS4. The pellets were suspended by pipetting the solution up and down, and 5 ml of the suspension were transferred into one of the two chambers of the labeled FLOTAC apparatus. Next, the FLOTAC apparatus was centrifuged for 5 min at 120 *g* in a Hettich Universal 320 centrifuge (Tuttlingen, Germany). Finally, after translation of the top portion of the FLOTAC apparatus, the observation grids of both chambers were examined for soil-transmitted helminth eggs at 100× magnification.

Fecal egg counts (FECs) for each helminth species were recorded separately for each Kato-Katz thick smear and each of the two FLOTAC observation grids. For quality control, 10% of the slides and observation grids were re-examined by a senior laboratory technician. In case the senior technician detected one or several eggs of a helminth species that had not been recorded in the original reading, the former result was considered as false-negative and replaced by the result of the senior technician. Moreover, in case of deviations in FECs of more than 10%, the original egg count was replaced by the result of the senior technician. In both cases, the microscopist was advised to read more carefully the following days. All Kato-Katz thick smears would have been re-read if there were discrepancies in the FECs in more than 20% of the re-examined slides, but this was never the case over the course of our study. The microscopists reading the FLOTAC observation grids were blinded to the results derived from the Kato-Katz method.

### Statistical analysis

Data were entered twice in Microsoft Excel version 10.0 (2002, Microsoft Corporation; Redmond, WA, USA) and checked for consistency with EpiData version 3.1 (EpiData Association; Odense, Denmark). Discrepancies were removed by consulting original data records. Data sets for Kato-Katz and FLOTAC results from the baseline screening and follow-up were merged by ID. Statistical analyses were carried out with STATA version 10 (StataCorp.; College Station, TX, USA).

For method comparisons, only individuals whose stool samples were subjected to duplicate Kato-Katz and FLOTAC at baseline *or* follow-up were included. We used the combined results of duplicate Kato-Katz and two FLOTAC observation grids as diagnostic ‘gold’ standard. A child with egg-positive microscopic test results in any Kato-Katz thick smear or FLOTAC observation grid was considered a true-positive. We assumed 100% specificity, and hence the complete absence of false-positive results for Kato-Katz and FLOTAC on the basis of unambiguously identifiable soil-transmitted helminth eggs under a microscope by experienced technicians. The sensitivity (proportion of true-positives detected by the test [26]) was calculated for duplicate Kato-Katz thick smears or the FLOTAC basic technique in relation to our ‘gold’ standard. The agreement between the results of the FLOTAC basic technique and duplicate Kato-Katz thick smear readings examined at baseline and follow-up was assessed for the diagnosis of *A. lumbricoides*, hookworm, and *T. trichiura* using kappa (κ) statistics [27]. Interpretation of κ statistics was as follows: <0.00 indicating no agreement, 0.00–0.20 indicating poor agreement, 0.21–0.40 indicating fair agreement, 0.41–0.60 indicating moderate agreement, 0.61–0.80 indicating substantial agreement, and 0.81–1.00 indicating almost perfect agreement [28]. The McNemar test was used to assess the inter-method differences in sensitivities, considering only individuals who were identified as positive for *A. lumbricoides*, hookworm, or *T. trichiura* according to the diagnostic ‘gold’ standard [29]. Statistical significance was given for *P*-values<0.05. The difference in intra-method sensitivity assessed before and after treatment was determined based on the assumption that non-overlapping 95% confidence intervals (CIs) indicate statistical significance.

Helminth-specific FECs of each individual were expressed as eggs per gram of stool (EPG), calculated by multiplying the sum of the two FECs from duplicate Kato-Katz thick smears by a factor 12. For FLOTAC, the FECs obtained from the two observation grids were added and multiplied by a factor (1/weight of stool sample) and expressed as EPG. The geometric mean (GM) EPG of the study cohort was calculated using the normal logarithm of the EPG plus 1 (GM = exp ((∑ log (EPG+1))/n)−1), where log (EPG+1) is the sum of the logarithm of each individual EPG, and one egg is added to each count to permit the calculation of the logarithm in case of EPG = 0 [30]. We calculated 95% CIs for sensitivity and the arithmetic mean (AM) EPGs and GM EPGs of the study cohort.

Participants with complete data from the baseline *and* follow-up survey, who received treatment and who were identified as positive for *A. lumbricoides*, hookworm, or *T. trichiura* according to our ‘gold’ standard at the baseline survey were included in the calculation of cure rate (CR) and egg reduction rate (ERR). The CR was determined as the percentage of children excreting eggs before treatment according to the ‘gold’ standard who became negative after treatment according to either the Kato-Katz or the FLOTAC method. CRs derived by the Kato-Katz or FLOTAC method were compared using a two-sample test of proportion. The ERR determined with Kato-Katz and FLOTAC from the treated children was calculated according to World Health Organization (WHO) guidelines [30], as follows: ERR = ((GM EPG before treatment – GM EPG after treatment)/GM EPG before treatment)×100. The group GM EPG used to determine the ERR was calculated from the group of individuals identified as positive for *A. lumbricoides*, hookworm, or *T. trichiura* according to the diagnostic ‘gold’ standard at the baseline survey.

## Results

### Operational results

**Figure 1:**
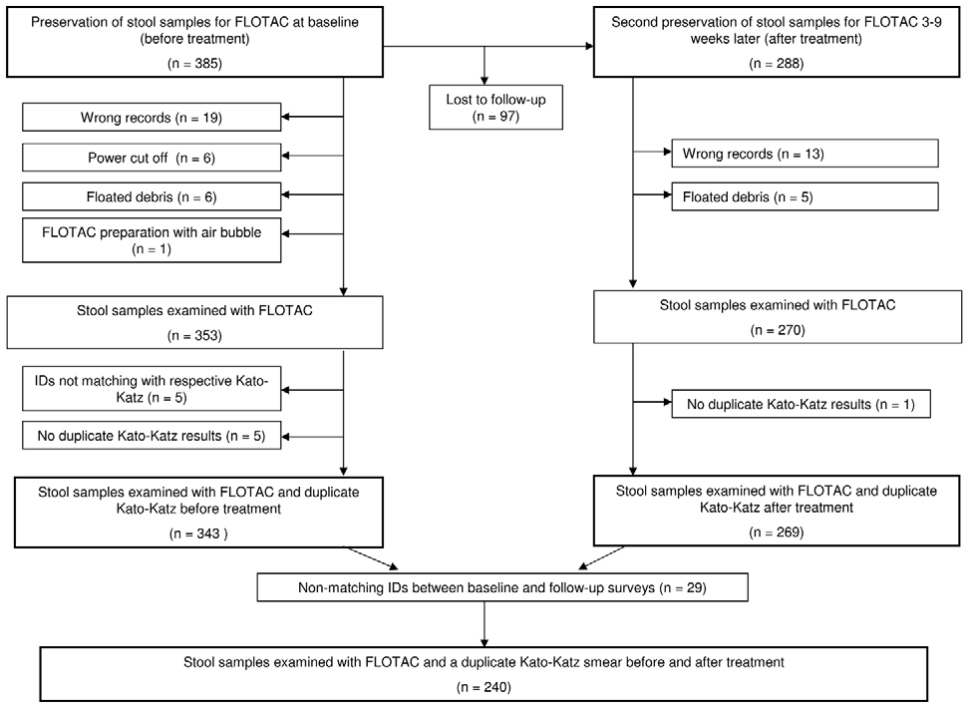
Number of stool samples examined with the Kato-Katz and FLOTAC method at baseline and follow-up. Flow chart detailing the data loss during stool preservation for FLOTAC, examination, data recording and matching FLOTAC results with duplicate Kato-Katz thick smear readings within the frame of a randomized controlled trial on anthelmintic drug efficacy and safety carried out in Zanzibar in early 2009.

Consent to participate in our trial was given by the parents and guardians of 1240 children, among whom 1066 children provided a stool sample at baseline. For FLOTAC examinations, 385 (36%) stool samples were preserved in 5% formaldehyde at baseline (Figure 1, left arm). Due to inaccurate preparation, a sudden power cut, and the flotation of stool debris, which hindered subsequent microscopic examinations, and because of erroneous labeling, the results from 32 stool samples preserved at baseline were not available. Additionally, five IDs did not match the IDs from the Kato-Katz results. For another five IDs only a single instead of duplicate Kato-Katz results were available. Hence, 343 among the 385 individuals (89%) had complete FLOTAC and duplicate Kato-Katz results at baseline. Among them 182 (53%) were girls and 161 (47%) boys. The age ranged between 6 and 20 years with a median of 11 years.

A second stool sample was preserved from 288 among the 385 individuals (75%) 3–9 weeks after the collection of the first stool sample (Figure 1, right arm). Among them 204 children were given one of four different anthelmintic treatments. Results from 18 preserved stool samples were lost due to the flotation of debris or incorrect labeling. Hence, 270 preserved stool samples were examined with the FLOTAC basic technique at follow-up. Complete FLOTAC and duplicate Kato-Katz results were available from 269 (70%) individuals at follow-up.

Since 29 IDs from the first and second stool examination data set did not match, complete examination data from the baseline and follow-up survey were available from 240 among the 385 originally selected study participants (62%). Among them, 174 were given anthelmintic drugs.

### Method comparison: diagnostic sensitivity

Results presented in Tables 1 and 2 show that the FLOTAC basic technique detected *T. trichiura* and *A. lumbricoides* infections with a higher sensitivity than duplicate Kato-Katz thick smears, but was less sensitive in detecting hookworm eggs, both at baseline and follow-up. At baseline, the sensitivity of FLOTAC for the diagnosis of *T. trichiura*, *A. lumbricoides* and hookworm was 95.0%, 87.5% and 54.1%, respectively, whereas the respective sensitivity of Kato-Katz was 88.0%, 67.5% and 81.1%. At follow-up, the sensitivity of FLOTAC for the diagnosis of *A. lumbricoides*, *T. trichiura* and hookworm was 97.4%, 93.3% and 61.2%, respectively, whereas the sensitivity of Kato-Katz was 41.7%, 84.9% and 77.6%, respectively.

**Table 1:** Diagnostic accuracy of duplicate Kato-Katz thick smears and the FLOTAC basic technique at baseline.

		*T. trichiura*			Hookworm			*A. lumbricoides*		
		n pos/EPG	%	95% CI	n pos/EPG	%	95% CI	n pos/EPG	%	95% CI
**Prevalence**	Kato-Katz	213	62.1	(56.9–67.3)	60	17.5	(13.5–21.5)	27	7.9	(5.0–10.7)
	FLOTAC	230	67.1	(62.1–72.1)	40	11.7	(8.3–15.1)	35	10.2	(7.0–13.4)
	‘Gold’ standard	242	70.6	(65.7–75.4)	74	21.6	(17.2–25.9)	40	11.7	(8.3–15.1)
**Kato-Katz**	Lower quartile (25%)	0			0			0		
**(2 slides)**	Median	36.0			0			0		
	Upper quartile (75%)	156.0			0			0		
	Arithmetic mean	235.2		(153.9–316.5)	43.6		(20.2–67.0)	970.4		(257.0–1683.8)
	Geometric mean	18.9		(14.2–25.2)	1.2		(0.8–1.7)	0.9		(0.5–1.3)
**FLOTAC**	Lower quartile (25%)	0			0			0		
**(2 chambers)**	Median	11.3			0			0		
	Upper quartile (75%)	60.0			0			0		
	Arithmetic mean	75.6		(47.7–103.5)	3.1		(0.2–5.9)	101.5		(10.1–193.0)
	Geometric mean	9.7		(7.6–12.3)	0.3		(0.2–0.4)	0.6		(0.3–0.9)
**Sensitivity**	Kato-Katz		88.0	(84.6–91.5)^a^^,^ b		81.1	(76.9–85.2)^c^^,^ d		67.5	(62.5–72.5)^e^^,^ f
	FLOTAC		95.0	(92.7–97.3)		54.1	(48.8–59.3)		87.5	(84.0–91.0)

Prevalence, quartiles, arithmetic mean and geometric mean eggs per gram of stool (EPG), and sensitivity with 95% confidence intervals (CI), as determined from stool samples examined with duplicate Kato-Katz thick smears and the FLOTAC basic technique at baseline in 343 school children from Kinyasini and Kilombero primary schools, Zanzibar, in March 2009. The diagnostic ‘gold’ standard was derived by the combined results of duplicate Kato-Katz thick smears and the FLOTAC basic technique.

a Differences in sensitivities determined by the McNemar test on positive individuals: *P* = 0.012.

b κ measure of agreement taking into account positive and negative individuals: κ = 0.74.

c Differences in sensitivities determined by the McNemar test on positive individuals: *P* = 0.006.

d κ measure of agreement taking into account positive and negative individuals: κ = 0.44.

e Differences in sensitivities determined by the McNemar test on positive individuals: *P* = 0.098.

f κ measure of agreement taking into account positive and negative individuals: κ = 0.68.

**Table 2:** Diagnostic accuracy of duplicate Kato-Katz thick smears and the FLOTAC basic technique at follow-up.

		*T. trichiura*			Hookworm			*A. lumbricoides*		
		n pos/EPG	%	95% CI	n pos/EPG	%	95% CI	n pos/EPG	%	95% CI
**Prevalence**	Kato-Katz	146	54.0	(48.3–60.3)	36	13.4	(9.3–17.5)	5	1.9	(0.6–4.3)^e^
	FLOTAC	154	57.3	(51.3–63.2)	30	11.2	(7.4–14.9)	11	4.1	(2.1–7.2)^e^
	‘Gold’ standard	165	61.3	(55.5–67.2)	49	18.2	(13.6–22.9)	12	4.5	(2.3–7.7)^e^
**Kato-Katz**	Lower quartile (25%)	0			0			0		
	Median	12.0			0			0		
	Upper quartile (75%)	90.0			0			0		
	Arithmetic mean	138.3		(90.6–186.0)	31.1		(4.0–58.1)	168.0		(0–341.5)
	Geometric mean	10.5		(7.5–14.6)	0.8		(0.5–1.2)	0.2		(0.01–0.3)
**FLOTAC**	Lower quartile (25%)	0			0			0		
	Median	2.7			0			0		
	Upper quartile (75%)	18.0			0			0		
	Arithmetic mean	35.6		(22.1–49.1)	1.4		(0.1–2.7)	78.2		(0–201.8)
	Geometric mean	4.4		(3.3–5.7)	0.2		(0.1–0.3)	0.2		(0.03–0.3)
**Sensitivity**	Kato-Katz		84.9	(80.6–89.1)^a^^,^ b		77.6	(72.6–82.5)^c^^,^ d		41.7	(35.8–47.6)^f^^,^ g
	FLOTAC		93.3	(90.4–96.3)		61.2	(55.4–67.1)		97.4	(95.4–99.3)

Prevalence, quartiles, arithmetic mean and geometric mean eggs per gram of stool (EPG), and sensitivity with 95% confidence intervals (CI), as determined from stool samples examined with duplicate Kato-Katz thick smears and the FLOTAC basic technique at follow-up in 269 school children from Kinyasini and Kilombero primary schools, Zanzibar, in May 2009. The diagnostic ‘gold’ standard was derived by the combined results of duplicate Kato-Katz thick smears and the FLOTAC basic technique.

a Differences in sensitivities determined by the McNemar test on positive individuals: *P* = 0.030.

b κ measure of agreement taking into account positive and negative individuals: κ = 0.73.

c Differences in sensitivities determined by the McNemar test on positive individuals: *P* = 0.201.

d κ measure of agreement taking into account positive and negative individuals: κ = 0.50.

e Binomial exact 95% confidence intervals.

f Differences in sensitivities determined by the McNemar test on positive individuals: *P* = 0.417.

g κ measure of agreement taking into account positive and negative individuals: κ = 0.49.

The inter-method sensitivity between FLOTAC and Kato-Katz differed significantly for the detection of *T. trichiura* at baseline and follow-up (*P* = 0.012 and *P* = 0.030) and for hookworm at baseline (*P* = 0.006). The intra-method sensitivity assessed at baseline and follow-up differed significantly for the diagnosis of *A. lumbricoides* only, according to non-overlapping 95% CIs.

Moderate-to-substantial agreement between the two diagnostic techniques was observed for all helminths investigated, before and after treatment. The highest agreement (κ = 0.74) was observed for *T. trichiura* both before and after treatment, whereas the lowest agreement was noted for hookworm diagnosis at baseline (κ = 0.44).

### Observed prevalence and infection intensities

In line with a higher sensitivity of the FLOTAC basic technique for the diagnosis of *A. lumbricoides* and *T. trichiura*, the observed prevalences of *T. trichiura* and *A. lumbricoides* infections determined with FLOTAC were higher compared to the ones derived by the Kato-Katz method. The opposite was observed for hookworm. At baseline, *T. trichiura*, hookworm and *A. lumbricoides* infections were detected in 67.1%, 11.7% and 10.2% of the children, respectively, when using FLOTAC. The respective prevalences according to Kato-Katz were 62.1%, 17.5%, and 7.9% (Table 1). Considering the results from the two methods combined, the respective prevalences were 70.6%, 21.6%, and 11.7%. The GM EPGs revealed with the Kato-Katz method were higher than those obtained with FLOTAC, showing values of 18.9 EPG for *T. trichiura*, 1.2 EPG for hookworm, and 0.9 EPG for *A. lumbricoides vs.* 9.7 EPG, 0.3 EPG and 0.6 EPG, respectively.

At follow-up, after 193 out of the 269 children (72%) had received experimental treatment, observed prevalences of *T. trichiura*, hookworm and *A. lumbricoides* infections had decreased to 57.3%, 11.2% and 4.1%, respectively, according to the FLOTAC technique. The respective prevalences according to the Kato-Katz method were 52.0%, 14.1% and 1.9% (Table 2). Results of both methods combined revealed prevalences of 61.3%, 18.2% and 4.5%, respectively. As expected, the GM EPGs were lower at follow-up than at baseline. The Kato-Katz method revealed GM EPGs of 10.5 EPG, 0.8 EPG and 0.2 EPG for *T. trichiura*, hookworm and *A. lumbricoides*, respectively. The FLOTAC method revealed respective GM EPGs of 4.4 EPG, 0.2 EPG and 0.2 EPG.

### Estimated CR and ERR

Table 3 shows diagnostic method-specific CRs and ERRs estimated for those children who were treated, had complete data records and were identified as positive for *A. lumbricoides*, hookworm, or *T. trichiura* according to the ‘gold’ standard at the baseline survey. Employing duplicate Kato-Katz thick smears before and after treatment revealed CRs of 91.3%, 61.2% and 41.4% against *A. lumbricoides*, hookworm and *T. trichiura* infections, respectively. The estimated CRs using FLOTAC were lower for *A. lumbricoides* (82.6%) and *T. trichiura* (36.2%), but higher for hookworm (69.4%). However, none of the differences showed statistical significance. The ERRs determined with the Kato-Katz method for *A. lumbricoides*, hookworm and *T. trichiura* infections were 99.9%, 89.9% and 87.6%, respectively, and with the FLOTAC method 99.4%, 65.5% and 80.7%, respectively.

**Table 3:** Drug efficacy as determined with the Kato-Katz method and FLOTAC basic technique.

		*T. trichiura*			Hookworm			*A. lumbricoides*		
		n pos/EPG	%	95% CI	n pos/EPG	%	95% CI	n pos/EPG	%	95% CI
**Baseline**										
**Prevalence**	Kato-Katz	174	100		41	83.7	(73.0–94.4)	19	82.6	(65.9–99.4)
	FLOTAC	163	93.7	(90.0–97.3)	25	51.0	(36.5–65.5)	22	95.7	(86.6–100)
	‘Gold’ standard	174	100		49	100		23	100	
										
**GM**	Kato-Katz	121.4		(98.3–149.7)	42.6		(22.4–80.5)	1119.2		(220.7–5659.6)
	FLOTAC	29.7		(22.9–38.4)	2.0		(1.0–3.5)	193.7		(61.9–601.6)
**Follow-up**										
**Prevalence**	Kato-Katz	102	58.6	(51.2–66.0)	19	38.8	(24.6–52.9)	2	8.7	(0–21.2)
	FLOTAC	111	63.8	(56.6–71.0)	15	30.6	(17.2–44)	4	17.4	(6.3–34.2)
**GM**	Kato-Katz	15.1		(10.0–22.5)	4.3		(1.8–9.3)	1.2		(0–5.8)
	FLOTAC	5.7		(4.1–7.8)	0.7		(0.3–1.2)	1.1		(0–4.1)
										
**CR**	Kato-Katz		41.4	(34.1–48.7)^a^		61.2	(47.6–74.9)^b^		91.3	(79.8–100)^c^
	FLOTAC		36.2	(29.1–43.3)		69.4	(56.5–82.3)		82.6	(67.1–98.1)
										
**ERR (GM)**	Kato-Katz		87.6			89.9			99.9	
	FLOTAC		80.7			65.5			99.4	

Prevalence, geometric mean (GM) eggs per gram of stool (EPG), cure rate (CR) and egg reduction rate (ERR), as determined from stool samples examined with duplicate Kato-Katz thick smears and the FLOTAC basic technique in relation to the diagnostic ‘gold’ standard from school children treated with anthelmintic drugs. The diagnostic ‘gold’ standard was derived by the combined results of duplicate Kato-Katz thick smears and the FLOTAC basic technique.

a Two-sample test of proportions: *P* = 0.322.

b Two-sample test of proportions: *P* = 0.396.

c Two-sample test of proportions: *P* = 0.381.

A total of 66 children had a stool sample examined with duplicate Kato-Katz thick smears and the FLOTAC basic technique at baseline and follow-up, without treatment in between. Among them, eight, seven and four children were identified to be infected with *T. trichiura*, hookworm and *A. lumbricoides*, respectively, by the Kato-Katz method at baseline (Table 4). The FLOTAC technique identified 22, five and four positive children, respectively. At follow-up, 24 and nine children were infected with *T. trichiura* and hookworm, respectively, according to the Kato-Katz method. The FLOTAC technique identified 26 children with a *T. trichiura* infection, 10 with hookworm eggs in their stool and one case of *A. lumbricoides*.

**Table 4:** Infection characteristics in 66 untreated children at baseline and follow-up.

		*T. trichiura*			Hookworm			*A. lumbricoides*		
		n pos/EPG	%	95% CI	n pos/EPG	%	95% CI	n pos/EPG	%	95% CI
**Baseline**										
**Prevalence**	Kato-Katz	8	12.1		7	10.6		4	6.1	
	FLOTAC	22	33.3		5	7.6		4	6.1	
**GM**	Kato-Katz	0.8		(0.2–1.7)	0.6		(0.1–1.2)	0.4		(0–0.9)
	FLOTAC	1.8		(0.8–3.3)	0.1		(0–0.2)	0.2		(0–0.5)
**Follow-up**										
**Prevalence**	Kato-Katz	24	36.4		9	13.6		0	0	
	FLOTAC	26	39.4		10	15.2		1	1.5	
**GM**	Kato-Katz	4.2		(2.0–8.2)	0.8		(0.2–1.7)	0		
	FLOTAC	2.1		(1.0–3.6)	0.3		(0.1–0.5)	0.04		(0–0.1)

Prevalence and geometric mean (GM) eggs per gram of stool (EPG), as determined from stool samples examined with duplicate Kato-Katz thick smears and the FLOTAC basic technique at baseline and follow-up from 66 school children not treated with anthelmintic drugs.

## Discussion

We found a significantly higher sensitivity of the FLOTAC basic technique compared to the Kato-Katz method for the diagnosis of *T. trichiura*, both before and after anthelmintic drug administration. The sensitivity of FLOTAC was also higher for the diagnosis of *A. lumbricoides* at both time points, but the difference showed no statistical significance. With regard to hookworm diagnosis, FLOTAC showed a significantly lower sensitivity before experimental chemotherapy. The intra-method sensitivity assessed before and after treatment showed considerable heterogeneity for *A. lumbricoides* diagnosis, but not for the other two soil-transmitted helminth species investigated, notwithstanding significantly lower FECs after treatment for each species. In general, the GM EPGs obtained with the Kato-Katz method were several-fold higher than those derived from the FLOTAC method. ERRs determined by FLOTAC after anthelmintic treatment were lower than those derived by the Kato-Katz method. There was no statistically significant difference in the CRs as determined by the Kato-Katz or FLOTAC method. However, the FLOTAC revealed somewhat lower CRs than the Kato-Katz method for both *T. trichiura* (36% *vs.* 41%) and *A. lumbricoides* (83% *vs.* 91%). The opposite was found for hookworm (69% *vs.* 61%).

The higher sensitivity of the FLOTAC basic technique for *A. lumbricoides* and *T. trichiura* diagnosis compared to the Kato-Katz method is in line with previous studies [21], [22]. The lower sensitivity for detecting hookworm eggs reported here, however, is in contrast to prior investigations performed with stool samples from Côte d'Ivoire and Zanzibar [19], [21], [22]. In our hands now, the sensitivity of FLOTAC for hookworm diagnosis was as low as 54% at baseline and slightly higher at follow-up (61%), whereas in the previous studies, sensitivities above 80% were reported [19], [21]. Four issues are offered for consideration, which might explain these observations. First, in the current study, we rigorously adhered to examining Kato-Katz thick smears within 20–40 min after preparation for hookworm egg counts to avoid over-clearance due to glycerol-soaked cellophane strips [31]. This had likely benefited the sensitivity outcome of Kato-Katz. Indeed, a limitation of our previous studies had been that we examined the slides for hookworm eggs only after 40–60 min post-preparation, which might have resulted in hookworm egg over-clearance [19], [21].

Second, the stool samples in the previous studies were preserved in sodium acetate-acetic acid-formalin (SAF), whereas in the current study 5% formaldehyde was used. A potential negative impact of the stool preservation media and FS on fragile hookworm eggs have been discussed before [18], [20], [22].

Third, the higher sensitivity of FLOTAC for hookworm diagnosis at follow-up compared to baseline is pointing to a negative impact of the duration of stool preservation on hookworm eggs (samples collected at follow-up had at least a 3-week shorter preservation period than samples preserved at the baseline survey), which is in line with findings from Côte d'Ivoire [20], [22].

Fourth, floated organic debris might have averted the accurate detection of transparent hookworm eggs in some of our stool samples, and hence negatively impacted on the sensitivity of FLOTAC for hookworm diagnosis. This latter problem was recently observed in stool samples collected from school children in Côte d'Ivoire and Pemba island, where it was overcome by including a washing step with ether or ethyl acetate to remove the organic debris or by a higher dilution of stool samples using tap water [22], [23].

The comparable sensitivities of either method at baseline and follow-up, despite a considerable decrease in FECs, suggest that a decrease in sensitivity only occurs if FECs fall under the lower detection limit of a method (i.e., 12 EPG for duplicate Kato-Katz thick smears, 24 EPG for a single Kato-Katz thick smear and 1 EPG for the FLOTAC basic technique). This suggestion is supported by the finding that those seven individuals found *A. lumbricoides*-positive by FLOTAC, but not with duplicate Kato-Katz thick smears at follow-up showed FECs of 9.8 EPGs and below, which likely explains the significant difference in the intra-method sensitivity for *A. lumbricoides* diagnosis before and after treatment.

The considerably lower numbers in the GM EPGs of our study cohort derived by FLOTAC in comparison to Kato-Katz are in line with previous studies [19], [21], [22]. Since there is no evidence of an upper detection limit of eggs of the FLOTAC method or of an artificial distortion in FECs associated with the smaller amount of biological material examined with the Kato-Katz method, but rather a linear relationship between FECs detected by the Kato-Katz and FLOTAC method, the following two hypotheses are offered for consideration. First, the amount of fecal material used in the Kato-Katz template (41.7 mg) is filtered, which might act like a concentration step, and hence contains a higher number of eggs [32], [33], whereas the amount of stool used for FLOTAC (∼1 g) is measured before filtering and contains heavy fibers, seeds and other undigested foodstuffs, but there is no concentration of eggs. Second, the FLOTAC procedure might not bring all helminth eggs into flotation in the FS-stool suspension, but only a certain proportion. Hence, some eggs would remain in the stool debris pellet. This might happen due to a variety of reasons, including the physical damage of eggs or slightly different densities of fertilized and unfertilized eggs. Clearly, additional investigations are warranted to elucidate these hypotheses.

The somewhat lower observed CRs against *T. trichiura* and *A. lumbricoides* and the lower ERRs of all soil-transmitted helminth infections identified by FLOTAC requires further study, as it might strategically impact on future helminth control programs. For example, if one considers that anthelmintic drug efficacy is lower than generally assumed, one might conclude that preventive chemotherapy fails to bring prevalence and infection intensities to sufficiently low levels, and hence more emphasis should be placed on other preventive measures, such as health education, the implementation of sewage systems, and improving sanitation and access to clean water. Additionally, in case anthelmintic drugs are less efficacious than assumed, then the risk of resistance development is likely higher than expected, since a larger proportion of helminths survive chemotherapy, which might select for resistant strains [34].

Before generalizing these results one must consider, however, that our study design suffers from the following shortcomings: (i) the study was not adequately powered for clinically important findings; (ii) CRs and ERRs were estimated only for individuals who were found *T. trichiura*-positive by the Kato-Katz method at baseline and occasionally co-infected with *A. lumbricoides* or hookworm; (iii) the number of individuals infected with *A. lumbricoides* or hookworm at baseline and treated with anthelmintic drugs was low; (iv) only a single stool sample was examined per person at baseline and follow-up with the FLOTAC basic technique, therefore not accounting for day-to-day variation in helminth egg output [35]; and (v) perfect specificities were assumed for the Kato-Katz and FLOTAC method. Hence, the current sampling scheme, dictated by the primary outcome measure of the randomized controlled trial (i.e., drug efficacy against *T. trichiura*) [24] might have biased our results and points (iv) and (v) might have resulted in an inaccurate estimate of the test sensitivity [14], [36].

There are several causes for the high loss of samples from the baseline to the follow-up survey. First, 97 children did not submit a stool sample at follow-up. Among them 87 (89.7%) were from the *T. trichiura*-negative, and hence non-treatment group, which we did not follow as rigorously as the treated children who were part of a randomized controlled trial [24]. Second, ∼1% of the stool samples could not be analyzed due to the flotation of stool debris directly under the examination grid of the FLOTAC apparatus. Third, another 1% of the samples were lost due to a sudden power cut. Fourth, a considerable number of results was not recorded or lost due to inappropriate labeling of the tubes, the FLOTAC apparatus or the Kato-Katz slides. Points two and three constitute limitations of the FLOTAC technique that should be taken into consideration in future studies. The flotation of debris can be overcome by an additional ether washing step of the stool sample to remove organic compounds. The ether washing step results in a more clearly examinable grid of the FLOTAC apparatus and improves the detection of *A. lumbricoides* and *T. trichiura* eggs [22]. It seems, however, to impact negatively on the detection of hookworm eggs [22], and hence a more appropriate way to lower the contamination of the FLOTAC examination grid has to be found. The problems of sudden power cuts in resource-constrained countries can be overcome by the use of mirror-operated microscopes and by hand-operated centrifuges [37]. Of course, these options are more laborious and time consuming, and can hence not be considered as ideal solutions for large-scale surveys. Point four implicates human failure. Since five labeling or recording steps (i.e., preservation tube, weight records, centrifugation tube, apparatus, and result records) are needed for FLOTAC, but only two (i.e., slide and result records) for Kato-Katz, the FLOTAC method is more error-prone, especially if large numbers of stool samples are processed under time constraints. In general, the application of the FLOTAC technique is more complicated and expensive than the Kato-Katz method [38]. This needs to be considered when applying the FLOTAC in field laboratories and large-scale epidemiological surveys, where ease of examination is beneficial.

While it is still too early to generalize the results reported here, and the FLOTAC technique might need further optimization for reliable diagnosis of hookworm infections, this new copro-microscopic technique holds promise for simultaneous detection of the three common soil-transmitted helminths, *S. mansoni* and intestinal protozoa infections [21], [22]. If these issues are solved, we are confident that the FLOTAC can serve as a viable alternative to the Kato-Katz method for anthelmintic drug efficacy assessment and for monitoring and evaluation of deworming programs, particularly in settings where infections intensities have come down to low levels after repeated treatment. The lower CRs and ERRs identified by FLOTAC warrant more investigation and, if confirmed, could strategically impact on future helminth control programs.

## Supporting Information

Alternative Language Abstract S1 Diagnostische Genauigkeit der Kato-Katz und FLOTAC Methode bei der Bestimmung Anthelminthischer Medikamentenwirksamkeit - Translation of abstract into German by Stefanie Knopp.
(0.03 MB DOC)

Checklist S1 STARD checklist.
(0.05 MB DOC)

Protocol S1 Study protocol of the randomized controlled trial, which provided the base of our study on diagnostic accuracy of the Kato-Katz and FLOTAC techniques for assessing anthelmintic drug efficacy.
(0.51 MB DOC)
