# Supplementary material for: Diagnostic Accuracy of Kato-Katz and FLOTAC for Assessing Anthelmintic Drug Efficacy
Source: PLoS Negl Trop Dis. 2011 Apr 12;5(4):e1036. doi: 10.1371/journal.pntd.0001036 (PMC3075226; doi:10.1371/journal.pntd.0001036)
Supplement: Alternative Language Abstract S1 — Diagnostische Genauigkeit der Kato-Katz und FLOTAC Methode bei der Bestimmung Anthelminthischer Medikamentenwirksamkeit - Translation of abstract into German by Stefanie Knopp. (0.03 MB DOC) [file pntd.0001036.s001.doc]

**Diagnostic Accuracy of Kato-Katz and FLOTAC for Assessing Anthelmintic Drug Efficacy:**

**Diagnostische Genauigkeit der Kato-Katz und FLOTAC Methode bei der Bestimmung Anthelminthischer Medikamentenwirksamkeit**

**Zusammenfassung**

***Hintergrund:*** Diagnostische Methoden mit hoher Sensitivität werden zur genauen Bestimmung der Prävalenz und Intensität von Helminthen-Infektionen in Gegenden, in denen die Bevölkerung regelmässig entwurmt wird, und zur Überprüfung der Wirksamkeit von Medikamenten, die zur Entwurmung eingesetzt werden, benötigt. Hier vergleichen wir die diagnostische Genauigkeit der Kato-Katz Methode und der FLOTAC Technik im Rahmen einer Studie zur Wirksamkeit von Anthelminthika, die im Jahr 2009 in Sansibar, einer Insel vor Tansania, durchgeführt wurde.

***Methoden/wichtigste Ergebnisse:*** In einer ersten Massen-Untersuchung wurden Stuhlproben von 343 Kindern mit zweifachen Kato-Katz Ausstrichen und der FLOTAC Technik untersucht. Die FLOTAC Technik hatte eine höhere Sensitivität als die Kato-Katz Methode für die Diagnose von *Trichuris trichiura* (95% *vs.* 88%, p = 0.012) und *Ascaris lumbricoides* (88% *vs.* 68%, p = 0.098), aber eine niedrigere Sensitivität für die Erkennung von Hakenwurm Eiern (54% *vs.* 81%, p = 0.006). Wenn wir die Ergebnisse beider Methoden als diagnostischen ‘Gold’ Standard zusammenfassen, waren die Prävalenzen von *T. trichiura*, Hakenwurm und *A. lumbricoides* 71% (95% Vertrauensintervall (CI): 66-75%), 22% (95% CI: 17-26%) und 12% (95% CI: 8-15%). In einer Anschlussuntersuchung, 3-5 Wochen nachdem 174 der 269 wiederholt untersuchten Kinder mit anthelminthischen Medikamenten behandelt worden waren, konnten wir Heilungsraten von 91% (95% CI: 80-100%), 61% (95% CI: 48-75%) and 41% (95% CI: 34-49%) von *A. lumbricoides*, Hakenwurm and *T. trichiura* Infektionen beobachten, wenn die Kato-Katz Methode angewendet wurde. Die FLOTAC Methode offenbarte niedrigere Heilungsraten von *A. lumbricoides* (83%, 95% CI: 67-98%) und *T. trichiura* (36%, 95% CI: 29-43%), aber eine höhere Heilungsrate von Hakenwurm Infektionen (69%, 95% CI: 57-82%). Diese Unterschiede waren statistisch nicht signifikant. Der Vergleich der beiden Methoden zeigte beachtliche Unterschiede im geometrischen Mittel der im Stuhl gezählten Helminthen Eier. Die FLOTAC Technik offenbarte eine niedrigere Eireduktionsrate nach Behandlung als die Kato-Katz Methode.

***Schlussfolgerung/Bedeutung:*** Unsere Ergebnisse legen nahe, dass die FLOTAC Technik mit etwas Verbesserung als brauchbare Alternative zur Kato-Katz Methode in Studien zur Wirksamkeit von Medikamenten und zur Überwachung und Evaluation von Entwurmungsprogrammen eingesetzt werden könnte. Die niedrigeren Heilungs- und Eireduktionsraten, die mit der FLOTAC Technik ermittelt wurden, sollten näher in Betracht gezogen werden und könnten einen strategischen Einfluss auf zukünftige Programme zur Kontrolle von Helminthen-Infektionen haben.

***Übersetzung:*** Stefanie Knopp
