# Supplementary material for: Diagnostic Accuracy of Kato-Katz and FLOTAC for Assessing Anthelmintic Drug Efficacy
Source: PLoS Negl Trop Dis. 2011 Apr 12;5(4):e1036. doi: 10.1371/journal.pntd.0001036 (PMC3075226; doi:10.1371/journal.pntd.0001036)
Supplement: Protocol S1 — Study protocol of the randomized controlled trial, which provided the base of our study on diagnostic accuracy of the Kato-Katz and FLOTAC techniques for assessing anthelmintic drug efficacy. (0.51 MB DOC) [file pntd.0001036.s003.doc]

**Study protocol**

**Title**

Efficacy and safety of albendazole and mebendazole as single-dose or in combination with ivermectin against soil-transmitted helminth infections, particularly *Trichuris trichiura*, in schoolchildren in Zanzibar, Tanzania

**Institutions**

1. Swiss Tropical Institute, Department of Public Health and Epidemiology, P. O. Box, CH-4002 Basel, Switzerland;

2. Ministry of Health and Social Welfare, Zanzibar, Integrated Helminth Control Programme, P.O. Box 236, Zanzibar, United Republic of Tanzania;

3. Wolfson Wellcome Biomedical Laboratories, Department of Zoology, The Natural History Museum, Cromwell Road, London SW7 5BD, United Kingdom;

4. Department of Pathology and Animal Health, CREMOPAR Regione Campania, University of Naples “Federico II”, Via della Veterinaria 1, 80137 Naples, Italy

**Appendices**

Appendix 1: Study information and informed consent sheet

Appendix 2: Study participant information and consent sheet (Kiswahili)

Appendix 3: Detailed field and laboratory procedures

Appendix 4: Questionnaire on side effects

Appendix 4: Questionnaire on side effects

Appendix 5: Overview participation

Appendix 6: Overview laboratory results

Appendix 7: Overview laboratory results

Appendix 8: Flowchart of participants infected with T. trichiura and included in the RCT

# Introduction

## Investigators

**Stefanie Knopp (PhD Student; Co-investigator)**

Department of Public Health and Epidemiology

Swiss Tropical Institute

Socinstrasse 57

CH- 4002 Basel, Switzerland

Tel.: +41 284-8226

Fax: +41 61 284-8105

E-mail: s.knopp@unibas.ch

**Dr. Khalfan A. Mohammed (Epidemiologist and Programme Manager; local Principal-investigator)**

Helminth Control Laboratory Unguja

Ministry of Health and Social Welfare

P.O. Box 236

Zanzibar, Tanzania

Tel.: +255 777 432370

E-mail: kamsharjy@hotmail.com

**Prof. Jürg Utzinger (Epidemiologist and Project Leader; Co-investigator)**

Department of Public Health and Epidemiology

Swiss Tropical Institute

Socinstrasse 57

CH- 4002 Basel, Switzerland

Tel.: +41 61 284-8129

Fax: +41 61 284-8105

E-mail: juerg.utzinger@unibas.ch

**Dr. Hanspeter Marti (Parasitologist and Head of Diagnostic Unit; Co-investigator)**

Diagnostic Unit

Swiss Tropical Institute

Socinstrasse 57

CH- 4002 Basel, Switzerland

Tel: +41 61 284-8252

Fax: +41 61 284-8118

E-mail: hanspeter.marti@unibas.ch

**Prof. Giuseppe Cringoli (Veterinary and Parasitologist; Co-investigator)**

Department of Pathology and Animal Health

CREMOPAR Regione Campania

University of Naples “Federico II”

Via della Veterinaria 1

80137 Naples, Italy

Tel.: +39 081 253-6283

Fax: +39 081 253-6282

E-mail: cringoli@unina.it

**Prof. David Rollinson (Parasitologist, Co-investigator)**

Wolfson Wellcome Biomedical Laboratories

Department of Zoology

The Natural History Museum

Cromwell Road

London SW7 5BD, United Kingdom

Tel.: +44 20 7942 5181/5152

Fax: +44 20 7942 5518

E-mail: d.rollinson@nhm.ac.uk

# Background

## Helminth infections

Worm infections remain a major public health problem, particularly in tropical and subtropical regions, primarily affecting socio-economically deprived populations. It is estimated that over a third of the world’s population is currently infected with helminths (‘parasitic worms’) *(1,* 2). This proposal will focus on soil-transmitted helminths, which are estimated to infect more than a billion people globally and are highly prevalent across sub-Saharan Africa *(2,* 3).

Soil-transmitted helminths are intestinal parasitic worms. Humans are the definitive host. These parasites are rampant in areas where hygienic conditions are poor and environmental features are adequate (e.g. high temperatures, sufficient soil moisture). Infections frequently cause chronic and debilitating disease, mainly in infants, pre-school and school-aged children and pregnant women *(2, 4,* 5).

Humans become infected through ingestion of parasite eggs or penetration of infectious larvae through the skin (2). The adult soil-transmitted helminths can live for years in the human gastrointestinal tract. The most common soil-transmitted helminths are the large roundworm (*Ascaris lumbricoides*), the whipworm (*Trichuris trichiura*), and the hookworms (*Ancylostoma duodenale* and *Necator americanus*).

Morbidity due to soil-transmitted helminth infection and the rate of transmission are related to the number of worms harboured by the host (6). Generally, infections with soil-transmitted helminths have a negative impact on pregnancy and birth outcomes, hamper children’s cognitive and physical development, result in reduced work capacity, and therefore compromise the social and economic development of communities and entire nations *(3, 7,* 8). Recent estimates suggest that globally 807-1221 million people are infected with *A. lumbricoides*, 604-795 million with *T. trichiura*, and 576-740 million with hookworm *(1,* 2). Infections with multiple helminth species are common *(9,* 10). In Africa, it is currently estimated that 44% of soil-transmitted helminth infections are concentrated in just four countries, namely Nigeria, Democratic Republic of Congo, South Africa and Tanzania *(*11). The global burden of soil-transmitted helminthiasis is estimated at 39 million disability-adjusted live years (DALYs), hence similar to the global burden owing to malaria or tuberculosis *(7,* 12).

## Soil-transmitted helminthiasis in Zanzibar

In sub-Saharan Africa, more than 500 million people are infected with at least one species of soil-transmitted helminths *(1,* 3) and multiple species helminth infections are the norm rather than the exception *(9, 13-*15). Zanzibar, where the field work of the current project will be carried out is part of the United Republic of Tanzania and consists of two islands, Unguja and Pemba. On both islands soil-transmitted helminth infections were recognized as an important public health problem already in the early 1990s *(*16). Indeed 85% of the surveyed population and 99% of examined schoolchildren were infected with at least one of the major soil-transmitted helminths *(16-*18). Helminth control programmes have been launched in Zanzibar more than a decade ago, to combat soil-transmitted helminthiasis, schistosomiasis and lymphatic filariasis *(16, 19-*21). However, the burden of soil-transmitted helminthiasis remains elevated. Recent studies revealed an overall prevalence of 49.4% for soil-transmitted helminth infections in school-aged children from the 6 districts in Unguja Island *(*22) and a point-prevalence of 77% in all age groups in Kinyasini village *(*19). *T. trichiura* was found to be the predominant helminth species in school-aged children from all 6 districts of Unguja, reaching a point prevalence of 55.2% in district North A *(*22).

## Control of soil-transmitted helminth infections

In view of the ecological and human determinants governing the transmission of soil-transmitted helminths, the total pre-school and school-aged population in the World Health Organization (WHO) African Region is considered to be at-risk of infection (8). Additionally, pregnant women are a major risk group *(23,* 24). Periodic and repeated deworming with benzimidazoles (albendazole and mebendazole), complemented by basic sanitation and clean water, is considered the most cost-effective approach to control morbidity due to soil-transmitted helminth infections *(1, 12,* 25). The benefits of regular treatment in school-aged children include improvement of iron stores *(*26), growth and physical fitness *(26,* 27), and school attendance *(*28). Women that are treated once or twice during pregnancy are less anaemic, birth weight improved and infant mortality at 6 months decreased *(23, 29,* 30). Periodic deworming campaigns lead by the WHO have focused on schools, because the heaviest intensities of soil-transmitted helminth infections are most commonly encountered in school-aged children, and schools provide convenient and efficient infrastructure for delivery of helminth control interventions *(*31). A resolution urging member states to attain a minimum target of regular deworming of at least 75% and up to 100% of all at-risk school-aged children by 2010 was passed at the 54th World Health Assembly in May 2001 *(12,* 31). However, even if the removal of adult worms from the intestinal tract is the goal of anthelminthic treatment (2), the success of mass-drug administration in highly endemic regions is more accurately measured when evaluating infection intensities *(*32). Infection intensities are usually classified according to thresholds set forth by the WHO *(*33) in high, medium and low, expressed by the number of eggs per gram of stool (EPG). Infection intensities are regarded not only as indicator for the worm-burden in the host but also for the degree of environmental contamination with eggs and hence transmission.

The drugs of choice for the treatment of soil-transmitted helminth infections are albendazole and mebendazole. Due to their good safety profile with rare and generally mild and transient adverse events allows their use also by non-medical personnel, such as school teachers in school-based mass treatment campaigns *(*12). However, even if considered to be broad-spectrum anthelminthics, the drugs have different species-specific efficacies *(2,* 34). Whilst albendazole, administered in a single oral dose of 400 mg is highly efficacious against *A. lumbricoides* and hookworm infections, it is less efficacious in curing *T. trichiura* infections *(*35). The respective cure rates (CR) against these three helminths are 88%, 72% and 28% *(*34). Mebendazole in a single oral dose of 500 mg is slightly more efficacious than albendazole against *A. lumbricoides* and *T. trichiura* with observed CRs of 95% and 36%, respectively *(*34). Unfortunately mebendazole lacks efficacy in curing hookworm infections; the overall CR has been reported at 15% *(*34). Ivermectin is the first-line treatment in programmes to eliminate filarial worms (*Wuchereria bancrofti* and *Onchocerca volvulus*) but is not applied to combat soil-transmitted helminthiasis with the exception of *Strongyloides stercoralis* infections where it shows a good efficacy *(36,* 37). However, the combination of ivermectin and albendazole is reported to a higher efficacy in curing *T. trichiura* infections than the use of albendazole alone *(35,* 38), but in general the treatment of *T. trichiura* infections with the currently available anthelminthic drugs is unsatisfactory.

Rapid re-infection in areas of poverty and poor sanitation, hampers the sustainable control of soil-transmitted helminthiasis *(*10), and there is growing concern about the potential emergence of resistance through the widespread and frequent use of anthelminthics *(39,* 40). The identification of new anthelminthic drugs is hence of major importance.

Another problem in terms of accurate monitoring of soil-transmitted helminth control programmes and of the consistency of the efficacy of anthelminthic drugs is the low sensitivity of the parasitological diagnostic methods *(*40), e.g. of the widely applied Kato-Katz method *(*41). If infection intensities with soil-transmitted helminths are low (e.g. due to regular anthelminthic treatment regimens), this method often fails to detect cases due to the small amount of stool investigated *(42,* 43). Hence, the development of new and more sensitive diagnostic tools is of pressing necessity.

The recently developed FLOTAC method - a multivalent faecal egg-count technique - using up to 1 g of stool for investigation, shows a significantly higher sensitivity to detect helminth infections than the Kato-Katz method *(43,* 44). For an appropriate assumption of the CRs of drug efficacy trials, as well as for future monitoring of transmission control, the FLOTAC might become an important supportive diagnostic tool.

## Helminth control programmes in Zanzibar

Zanzibar serves as an example where long-term helminth control programmes have been implemented with both remarkable success and persistent problems. Studies carried out in Pemba and Unguja in the late 1980s and early 1990s revealed that the prevalence of helminth infections exceeded 85% in all surveyed population groups with a considerable amount of moderate, but only few heavy infection intensities *(16,* 18). Therefore, in 1992, a national plan of action for the control of helminths was implemented by the Ministry of Health and Social Welfare (MoHSW) *(*16). Periodical mass-treatment of schoolchildren with a single oral dose of mebendazole (500 mg) was adopted and portrayed as the most cost-effective strategy for the control of morbidity *(*16). As re-infection intensities were found to reach pre-treatment levels already 6 months after treatment, it was decided to treat the children every 4 to 6 months *(16,* 45). Finally, in 1994, the MoHSW established a national helminth control programme *(*46). Since then, large-scale preventive chemotherapy interventions are regularly carried out at schools and occasionally in whole communities *(*19). Currently, on Unguja, the helminth control programme operates from the Helminth Control Laboratory Unguja (HCLU), which provides, when sufficient donor funds are available, the necessary infrastructure and resources for mass drug administration campaigns targeted mainly towards school-aged children *(*47). From 1995 onwards, schoolchildren were treated on a regular basis with mebendazole (500 mg). Mebendazole was replaced by albendazole (400 mg) in 2003. Additionally, in the frame of the Programme to Eliminate Lymphatic Filariasis, from 2001 onwards, the whole eligible population was treated annually with ivermectin (200 µg/kg) and albendazole (400 mg) *(*19).

Studies on the efficacy of mebendazole (single oral dose of 500 mg) conducted on Pemba Island, revealed cure rates 21 days post-treatment of 97% for *A. lumbricoides*, 23% for *T. trichiura* and 8% for hookworm in children in 1999. The egg-reduction rate achieved with mebendazole was 99% for *A. lumbricoides*, 81% for *T. trichiura* and 52% for hookworm in the same children from Pemba *(*48). A study on the efficacy of albendazole (triple oral dose of 400 mg), conducted in schoolchildren from Unguja island in 1994, revealed cure rates of 99% for *A. lumbricoides*, 43% for *T. trichiura* and 88% for hookworm three weeks post-treatment. The egg-reduction rates were 99% for *A. lumbricoides*, 92% for *T. trichiura* and 99% for hookworm *(*18).

In contrast to these findings, a recent study conducted in Unguja revealed albendazole-cure rates of only 42% and 25% for *A. lumbricoides* and *T. trichiura* infections, respectively *(*47), indicating a considerable decrease in efficacy of this drug especially against *A. lumbricoides* compared to preceding studies. A decrease in the efficacy of mebendazole against soil-transmitted helminths was reported from Zanzibar, too *(*48). These observations call for a close monitoring of the efficacy of the drugs used for mass drug administration on Zanzibar to early recognize drug resistance development in soil-transmitted helminthiasis.

Although a recent study carried out in 2 schools in Unguja in 2007 showed that prevalences and infection intensities of all soil-transmitted helminth species declined since the onset of the control programme in 1994, the prevalences of *T. trichiura*, hookworm, *A. lumbricoides* and *S. stercoralis* were still 46.6%, 21.6%, 16.9% and 10.2%, respectively *(*49).

## Safety of the benzimidazoles

On Zanzibar, multiple studies on the efficacy of mebendazole, albendazole, ivermectin and albendazole plus ivermectin have been conducted *(18-20, 45, 46, 48, 50,* 51). Actively assessed, no severe side effects were recorded for albendazole or ivermectin in a trial conducted in Unguja in 1994, and mild side effects were of transient nature for both treatments *(*18). Although not actively assessed, no adverse events were reported after mebendazole treatment in the week following the administration of anthelminthics in Pemba *(*48). No severe adverse reactions were reported from a trial conducted in Zanzibar in 2006 to assess the safety of triple drug co-administration (albendazole plus ivermectin plus praziquantel) *(*19). The combined treatment of albendazole and ivermectin is successfully and safely applied to treat the Zanzibari population since 2001 in the frame of the GPELF *(*20). In general, the side effects of albendazole and mebendazole, at the dosage recommended for deworming, have been described as negligible and self-limiting *(*52). However, even if the single applications of albendazole, mebendazole and ivermectin are reported to be safe, to our knowledge, there are no reports on the combined treatment of mebendazole and ivermectin. Hence, the safety of the latter drug combination needs to be confirmed.

## Significance

In Zanzibar, helminth control programmes distributing anthelminthic drugs on a regular basis to schoolchildren and the whole eligible population successfully reduced the burden of soil-transmitted helminthiasis. Prevalences and infection intensities declined by more than 70% in children attending Chaani and Kinyasini primary schools in 2007 compared to children visiting the same schools in 1994 *(*49)*.* The only exception found was for *T. trichiura* infections. For this species the prevalence in the two schools in Zanzibar remained remarkably high (46.6%). One possible explanation for the persistence of *T. trichiura* infections in Zanzibar is the low efficacy of single-dose benzimidazoles against this helminth. Hence, well conducted studies on the performance of e.g. drug-combinations for the treatment of *T. trichiura* infections are needed to reduce the transmission also of this helminth.

In settings like Zanzibar, where the prevalence of soil-transmitted helminthiasis is relatively high and where anthelminthic drugs are regularly administered to large parts of the population, the risk of resistance development in worms is considerable. Because only a few anthelminthic drugs are on the market and only few are currently in the pipeline, emergence and spread of resistance must be minimized *(*53). Studies to re-assess the efficacy of anthelminthic drugs that have been used for many years and appraisal of efficacy of drug combinations are warranted as results can guide policy decisions. The development and validation of tools for the sensitive detection, monitoring and thus prevention of emerging drug resistance is recommended by WHO *(*12).

We propose to assess the safety and efficacy of the combination treatment of albendazole plus ivermectin versus mebendazole plus ivermectin versus single-dose albendazole and single-dose mebendazole against soil-transmitted helminth infections and particularly against *T. trichiura* infections in Zanzibar. The following considerations are further motivating our proposal.

First, CRs and ERRs are unsatisfactory when *T. trichiura* infections are treated with albendazole or mebendazole alone. These drugs were applied in the past decade for mass drug administrations in Zanzibar. The application of albendazole in combination with ivermectin as distributed in the frame of the GPELF is reported to be more effective in curing *T. trichiura* infections *(*35) but still *T. trichiura* prevalences are high in Zanzibar. Mebendazole is reported to have higher CR and ERR than albendazole but a combination therapy of mebendazole and ivermectin to cure *T. trichiura* infections has - to our knowledge - not been investigated so far. Second, mebendazole and ivermectin show a good safety profile when used for mono-therapy but the safety of this drug combination has net yet been assessed. Third, it is possible that after seven years of application of albendazole in the programme to eliminate lymphatic filariasis and after five years of application of albendazole in school-based treatment programmes in Zanzibar, resistance against albendazole started to develop in soil-transmitted helminth species. This possibility has not yet been investigated. Therefore it is of utmost importance to re-assess the efficacy of albendazole, mebendazole and their combinations with ivermectin to guide policy recommendations by the government of Zanzibar and the WHO in direction of which drug regimen is most efficacious and safe to apply in control programmes with the aim to reduce soil-transmitted helminth transmission and to also effectively target *T. trichiura*. Forth, closely related to investigations on drug efficacy, the evaluation of the diagnostic potential of the FLOTAC method in terms of its applicability in local laboratories and in comparison to the Kato-Katz method will contribute to the development of new tools which are needed for a sensitive diagnosis of low-intensity helminth infections.

# Goal

Our overreaching goal is to assess the current efficacy and safety of the anthelminthic drugs and drug combinations, (i) albendazole, (ii) albendazole plus ivermectin, (iii) mebendazole and (iv) mebendazole plus ivermectin, against the major soil-transmitted helminth species prevalent in Zanzibar. These are namely *A. lumbricoides*, hookworm and *T. trichiura*. Three specific objectives are related to this goal.

## Objectives

1. Efficacy and safety of albendazole and mebendazole alone and in combination with ivermectin against *T. trichiura*: We will carry out a randomized controlled trial in Kinyasini and Chaani school with the aim to compare the efficacy and safety of a single-dose treatment with albendazole (400 mg), and single-dose treatment with mebendazole (500 mg), a combination treatment with albendazole (400 mg oral dose) plus ivermectin ( 200 µg/kg oral dose) and a combination treatment with mebendazole (500 mg oral dose) plus ivermectin (200 µg/kg oral dose) against *T. trichiura.* Efficacy against other soil-transmitted helminth infections will also be determined to find out the most effective drug regimen to fight trichuriasis in particular and soil-transmitted helminthiasis in general. These results will guide policy recommendations in an appropriate way.

2. Development of resistance against albendazole/mebendazole after several years of mass-administration: To determine the current efficacy of a single-dose treatment with albendazole (400 mg single oral dose) or mebendazole (500 mg single oral dose) against *A. lumbricoides* and hookworm, to compare it to the efficacy of the drugs reported from Zanzibar in the 1990s and to shed light on a potential rise of drug resistance of *A. lumbricoides* and hookworm in Zanzibar within the above mentioned randomized controlled trial in Kinyasini and Chaani school.

3. Comparison of FLOTAC and Kato-Katz method: To compare the performance of the recently developed FLOTAC method with the Kato-Katz method applied by ‘default’ to diagnose soil-transmitted helminth infections under local conditions with the aim to determine their strengths and shortcomings in parasitological surveys and drug efficacy trials.

# Study design

## Study type and measurement

The study will be designed as randomized controlled trial. The primary outcome measurements will be the CR (percentage of children with egg counts >0 before treatment who become negative after treatment; e.g. the reduction in prevalence in the participants following treatment, calculated on a per-species basis and not taking into account the influence of the treatment on any co-infections) and the ERR (the reduction in egg count in a measured quantity of feces following treatment in those not cured; calculations based on the arithmetic mean egg count of the Kato-Katz slides prepared before and after treatment) revealed with the Kato-Katz method and achieved by treatment with any drug regimen for any soil-transmitted helminth species. The CR will be calculated using the formula ((N^+^/n) – (N_3_^+^/n))/(N^+^/n), where N^+^ is the number of positive children at baseline, N_3_^+^ is the number of children positive 3-4 weeks after treatment, and n is the total number of children with stool samples from both day 0 and 3-4 weeks post-treatment. The ERR induced by the treatment will be calculated as 100(1 – exp (-D))%, where D will be the mean difference (D = log_e_(c_0_+1) – log_e_(c_1_+1), where c_0_ is the egg count before treatment and c_1_ the egg count after treatment) for a particular treatment.

## Study area and population surveyed

The study will be carried out on Unguja, the main island of the Zanzibar archipelago belonging to the United Republic of Tanzania. The island is ~87 km long with a surface area of ~1660 km^2^. The Zanzibar archipelago has a relatively high precipitation with a mean annual rainfall of 1955 mm with heaviest rains occurring in March-May. The average annual temperature ranges from 23°C to 28°C. The population of Unguja (623,000 inhabitants in 2002) is mainly settled in the densely populated West of the island. Zanzibar’s major economic sectors are based on agriculture, trade, industries and tourism.

The schools of Kinyasini and Chaani, located in the North district of Unguja, are selected for this study. Selection criteria included:

1. high prevalences of soil-transmitted helminths (*T. trichiura*: 42.9%; hookworm: 22.5%; *A. lumbricoides*: 16.5%) were revealed in children visiting these schools in a study in 2007;
2. a high number of participants (94%) submitted at least 1 stool sample for examinations in a study in 2007;
3. the schools are regularly visited by members of the HCLU for annual screenings and subsequent treatment of the schoolchildren in the frame of the national helminth control programme; hence, the children and teachers are familiar with the team and facilitate study procedures; and
4. the schools are about 40 km out of Zanzibar Town and easily accessible by project vehicles on tamarack roads, guaranteeing a fast transfer of equipment to the school and of stool samples back to the HCLU.
5. 500-2000 children are visiting each of the two schools and thus the calculated sample size of 2000 children to include in the study will be available.
6. both schools can provide empty classrooms that will serve for collection of stool containers without disturbing teaching; these empty rooms will also serve for treatment and observation of acute adverse events for one hour after treatment and for medical consultation in privacy on the day of treatment.

# Patient inclusion and exclusion and withdrawal criteria

## Patient inclusion criteria

1. Age ≥5 years, both sexes.
2. Submission of 1 stool sample of sufficient size to perform a total of 2 thick Kato-Katz smears at the baseline parasitological survey.
3. Submission of 1 stool sample of sufficient size to perform a total of 2 thick Kato-Katz smears at the day of treatment.
4. Infection with *T. trichiura* as determined with the Kato-Katz method at the baseline survey. Additionally, also children with *A. lumbricoides* and/or hookworm infections will be included in one of the 4 treatment arms.
5. For females, not pregnant, as verbally assessed by medical personnel on the day of treatment.
6. Absence of major systemic illnesses, as assessed by medical personnel on the day of treatment.
7. No anthelminthic treatment in the past 4 weeks, as verbally confirmed by the participant at the baseline survey.
8. Written informed consent by the parent/legal guardian.

## Patient exclusion criteria

1. No stool sample given at baseline survey or day of treatment.
2. No infection with *T. trichiura*, *A. lumbricoides* and/or hookworm at baseline
3. Subjects with fever or other signs of acute illness.
4. Subjects with severe neurological disorder.
5. Subjects with severe liver disorder, as assessed by medical personal with an abdominal examination on the day of treatment.
6. Pregnant schoolgirls.
7. Recent history of anthelminthic treatment.
8. Consent not given.

## Patient discontinuation and withdrawal criteria

Apart from falling under the exclusion criteria the children’s participation in this research study is entirely voluntary. The children may choose not to participate and withdraw their/their parents’ consent of participation at any time. The children will not be penalized in any way if they decide not to participate or to withdraw from this study.

# Study and treatment protocol

## Field procedures

The headmasters of the schools will be informed about the purpose and procedures of the study. Once informed consent is obtained, the objectives and procedures will be explained to the teachers, schoolchildren and their parents and written informed consent (see Appendix 1) by the participants’ parents/legal guardians will be obtained.

Field procedures are as follows. The study will be conducted between March and May 2009 (for exact protocol see Appendix 2). In an in-depth baseline survey before treatment, approximately 2000 children will be asked to submit 1 stool sample of sufficient size (at least 5 g) to perform the anticipated examinations. On the first day of the survey, about 200 participants will be given a plastic container and invited to bring a fresh stool specimen the following morning. Upon collection, a unique identification number will be attached to the container and demographic data will be collected (e.g., age and sex).

This procedure will be repeated over several days, entering ~200 additional schoolchildren every day up to a total of 2000 children. One of the inclusion criteria for the randomized drug trial is an infection with *T. trichiura* (see point 5.). We anticipate that with an expected prevalence of 25% of *T. trichiura* infections and with a compliance of 95% working for 2-3 weeks in Kinyasini and Chaani schools will suffice to obtain the required sample size of at least 540 children infected with *T. trichiura*. In addition those children diagnosed with *A. lumbricoides* and/or hookworm will also be attributed to one of the 4 treatment regimens, but their numbers are not included into the sample size calculations. Before the onset of the trial, each individual included into the trial will be physically examined by a medical assistant for exclusion criteria.

Once all samples have been collected and analysed, a randomized treatment list allocating the IDs of the ~540 children infected with *T. trichiura* and of the remaining children infected with *A. lumbricoides* and/or hookworm to one of the 4 treatment regimens will be prepared. On the day before treatment the selected children will be given another stool container and they will be asked to provide another stool sample the following morning. On the day of treatment the stool samples will be collected and all children’s weights will be recorded. The children will be allocated to one of four groups, as follows:

1. Single-dose albendazole group; children will receive one tablet of albendazole (400 mg) and a placebo resembling ivermectin (number of tablet according to weight).
2. Mebendazole group; children will receive a single dose of mebendazole (500 mg) and a placebo resembling ivermectin (number of tablet according to weight).
3. Albendazole plus ivermectin group; children will receive one tablet of albendazole (400 mg) plus ivermectin tablets according to their weight (200 µg/kg).
4. Mebendazole plus ivermectin group; children will receive one tablet of mebendazole (500 mg) plus ivermectin tablets according to their weight (200 µg/kg).

The participants will be asked to swallow the drugs with clean water and the health worker administering the drugs will check that the tablets have been swallowed by looking into the mouth and under the tongue. During the entire treatment phase and for one additional hour, a clinician will be present for supervising drug intake, monitoring and registering acute adverse events of the four drug regimens and to provide medical assistance where necessary. The medical assistant will be provided with a complete list of possible adverse events of the respective drug. In case of adverse events, appropriate medical action will be taken, e.g. administration of drugs for relief.

The children will be kept under medical supervision for one hour after treatment. The staff of the Public Health Centres (PHC) located nearest to the two schools (Upenja and Chaani) will be informed and undergo a pharmacovigilance training in the week before treatment. Additionally, the PHCs will be provided with first aid drugs and will remain open for 24 hours on the day of treatment. Children, parents and teacher will be informed that in case of any adverse reaction they can refer cases to the PHC at day and night time for 24 hours. Forty eight hours after treatment, solicited adverse events due to the treatment will be assessed by a previously tested questionnaire (see Appendix 3). The onset and duration of the side effects will be recorded and graded as follows:

- *mild:* present but not requiring any interference;
- *moderate:* (1) present and requiring medication for symptomatic relief as requested by the affected people or (2) present and interfering with normal daily activities; and
- *severe:* present and required medical intervention beyond symptomatic relief.

Three to four weeks after drug administration, treated children will be given a follow-up stool container and asked to bring a fresh stool specimen the following morning for a follow up of the treatment. Upon collection of this container the children will be handed out another container and invited to bring a second stool sample the next morning.

In the last week of the study all infected study participants will be treated free of charge with the current drug of choice albendazole (400 mg) against *A.* *lumbricoides*, *T.* *trichiura* and hookworm. The subjects infected with any soil-transmitted helminth but falling under the exclusion criteria (no. 4 and 7) will be offered a treatment after termination of their illness or after pregnancy.

## Laboratory procedures

All stool samples will be transferred to the HCLU on the day of collection, and will be examined by experienced laboratory technicians and the PhD-student. At the baseline screening, 2 Kato-Katz thick smears *(*41) from one stool sample per individual using 41.7 mg of stool will be prepared and examined quantitatively for the presence of hookworm eggs after a clearing time of 20-30 min, and subsequently for *A. lumbricoides* and *T. trichiura* eggs. The eggs of *A.* *lumbricoides*, hookworms and *T. trichiura* will be counted and recorded separately. From 250 randomly selected stool samples 1-2 g will be preserved in SAF for subsequent examination with the FLOTAC method *(*54).

From children included into the randomized controlled trial and thus providing another stool sample at the day of treatment and 2 consecutive stool samples for the follow up, a double Kato-Katz thick smear will be prepared and examined. At the days of follow up 1-2 g of stool, from those individuals examined with FLOTAC before treatment and who have been included in the trial, will again be preserved in SAF for subsequent examination with the FLOTAC method. All laboratory investigations will be blinded, e.g. the technicians examining the Kato-Katz slides or FLOTAC apparatus will not be aware of the treatment regimen of the participants. A random sample of 10% of the Kato-Katz thick smears will be re-examined by a Tanzanian senior laboratory technician for quality control.

# Assessment of benefits and risks for the study population

Benefits of participating in the study will be as follows. First, participants will receive free laboratory investigations for soil-transmitted helminthiasis and will be informed in person about their parasitological and clinical results. Second, the infected participants will receive free treatment. Anthelminthic treatment can improve the children’s general health status, including better uptake of nutrition, reduced anemia, and improved growth and cognitive development. Third, at population level, the assessment of a more effective treatment regimen against *T. trichiura* and the early detection of a potential resistance against certain anthelminthic drugs in the frame of this study can lead to appropriate policy recommendations by the Zanzibari government and the WHO and enhance the success of helminth control in Zanzibar and elsewhere.

Risks to participate in the study are due to the side effects that may be associated with anthelminthic drugs and that are generally reported to be mild, transient and self-limiting. Side effects can include fatigue, headache, dizziness, allergic reactions, shivering, abdominal pain, nausea, vomiting, diarrhea or fever.

Medical personal and the drivers of the helminth control team will remain with the treated study participants for one hour after treatment and in case of any acute adverse reaction refer the child to the nearest PHC. The PHCs will remain open for 24 hours on the day of treatment and will be provided with first aid drugs before treatment starts. Children, parents and teachers will be instructed that in case of any adverse reaction they can refer cases to the nearest PHC at day and night time for 24 hours after treatment. In case of any adverse reaction the participant will be treated free of charge.

# Data analysis

The following data will be available for each individual:

Name

Age

Sex

Date of treatment

Drug used

Date of follow up

Parasitological data of first stool examination (double Kato-Katz thick smear)

Parasitological data of second stool examination (double Kato-Katz thick smear and single FLOTAC)

Parasitological data of follow up (2 stool samples with 2 Kato-Katz thick smears per sample and single FLOTAC)

Data on adverse events of treatment after 3 h (acute) and 72 h (solicited).

All data will be entered twice in Microsoft Excel version 10.0 (2002 ® Microsoft Corporation) by local staff in Zanzibar. Datasets will be compared using EpiData version 3.1 (EpiData Association; Odense, Denmark), and discrepancies removed based on the original records by the PhD student (Stefanie Knopp) when back in Switzerland. Statistical analyses will be carried out by the PhD student with JMP version 5.0.1 (SAS Institute; Cary, NC, United States of America). The CR of the treatments and the ERR will be compared for each drug according to the results of either the Kato-Katz or the FLOTAC method or the combination of both methods.

In addition, the frequency and severity of acute and solicited adverse events of the different drug regimens will be compared. The latter analysis will try to seek differential effects according to age, sex and initial parasite load.

# Sample size

Sample size calculations are based on the differences in CR of albendazole or mebendazole versus albendazole and ivermectin against *T. trichiura*.

The CRs of treatment with albendazole (400 mg single oral dose) and mebendazole (500 mg single oral dose) have been reported in a recent meta-analysis to be 28% (95% CI, 13%-39%) and 36% (95% CI, 16%-51%) for *T. trichiura*; 88% (95% CI, 79%-93%) and 95% (95% CI, 91%-97%) for *A. lumbricoides*; and 72% (95% CI, 59%-81%) and 15% (95% CI, 1%-27%) for hookworm infections *(*34).

The CR of the combination treatment of albendazole (400 mg single oral dose) plus ivermectin (200-400 µg/kg) as single therapy were reported from Haiti and the Philippines for the major soil-transmitted helminths and indicated to be 79.6% and 65.1%, respectively for *T. trichiura*, 100% and 78.1%, respectively for *A. lumbricoides* and 100% for hookworm infections *(38,* 55).

No relevant references on a combination therapy of mebendazole and ivermectin exist according to a recent review on the efficacy and safety of drug combinations *(*35).

Information about CR and ERR of albendazole, mebendazole and ivermectin are also available for Zanzibar. The CRs of a single dose of albendazole (400 mg) and mebendazole (500 mg) were assessed in a single-blind randomized controlled trial performed in Pemba, Zanzibar, in 1992/93 and were 10.5% and 14.2% for *T. trichiura*, 98.9% and 97.8% for *A. lumbricoides* infections and 56.8% and 22.4% for hookworm *(*51). ERRs for albendazole and mebendazole in the same study were 73.3% and 81.6% for *T. trichiura*, 99.6% and 99.3% for *A. lumbricoides* infections and 97.7% and 82.4% for hookworm *(*51).

The CR and ERR of a single dose ivermectin (200 µg/kg) were assessed in Unguja in 1994 and were 11.3% and 58.9% for *T. trichiura*, 100% and 100% for *A. lumbricoides* infections and 0% and 0% for hookworm *(*18).

The sample sizes indicated in **Table 1** were calculated in Microsoft Excel with an equation given by Fleiss *(*56):

7.85*((A1*(1-A1))+(B1*(1-B1)))/(ABS(A1-B1))^2+2/ABS(A1-B1)+2

Where A1 is the estimated CR from treatment A (e.g. albendazole or mebendazole) and B1 is the estimated CR from treatment B (e.g. albendazole plus ivermectin or mebendazole plus ivermectin).

Using a significance level of 5% to detect greater efficacy of one treatment, and a power of 80%, the following table indicates the required number of individuals **per treatment group** at different CRs:

**Table 1: Cure rates for *T. trichiura***

**Treatment A (albendazole or mebendazole)**

| **Treatment B**  **(albendazole or mebendazole plus ivermectin)** | 15% | 20% | 30% | 35% | 40% | 45% | 50% | 55% |
| --- | --- | --- | --- | --- | --- | --- | --- | --- |
| 60% | 21 | 27 | 48 | 69 | 106 | 185 | 407 | 1573 |
| 65% | 17 | 21 | 36 | 48 | 69 | 105 | 182 | 395 |
| 70% | 14 | 18 | 28 | 36 | 48 | 67 | 102 | 175 |
| 75% | 12 | 15 | 22 | 27 | 35 | 47 | 65 | 97 |
| 80% | 10 | 12 | 18 | 21 | 27 | 34 | 44 | 61 |
| 85% | 9 | 10 | 14 | 17 | 21 | 25 | 32 | 41 |
| 90% | 8 | 9 | 12 | 14 | 16 | 20 | 24 | 29 |

Based on the given information we estimate the CR of albendazole/mebendazole for *T. trichiura* infections to be between 20% and 45% and the CR of the combination of albendazole/mebendazole plus ivermectin to be between 65% and 80%. Hence, a sample size of at least 105 participants per treatment group is needed for significance analysis.

In the proposed study sites, the prevalence of *T. trichiura* infections is most likely to be above 30% and the compliance is estimated to be around 90%. These assumptions are based on a study performed in Kinyasini and Chaani schools in 2007 where 25.9% of the children were found to be infected with *T. trichiura* after examination of 1 stool sample and 47.9% after examination of 3 stool samples with the Kato-Katz method and where the compliance to submit 1-3 stool samples was >85%. In view of the required number of cases per group, and allocating for patients lost to follow-up, at least 2000 subjects would have to be screened so that 500-600 infected individuals infected with *T. trichiura* can enter the trial (see Appendix 5).

# Ethical considerations

The study protocol will be reviewed by the institutional research commission of the STI, and the institutional review board of the World Health Organization (WHO). After ethical approval by the Ethikkommission beider Basel (EKBB) the study protocol will be submitted for ethical clearance to the Ministry of Health and Social Welfare (MoHSW) of Zanzibar.

The headmasters of Kinyasini and Chaani schools will be informed about the aim of the study. The study participants will be given detailed information about the purpose of the study, extend of their involvement (stool collection, questionnaire about side effects) and their right to be treated free of charge if found positive. Written informed consent will be obtained from the parents/legal guardians of the participating children. It will be pointed out that participation is voluntary and that the individuals may withdraw from the trial at any time. At the end of the study the participants will be informed about the result of their stool examination and those found positive will be treated by a member of HCLU with respective to their infection and at recommended doses and following standard treatment schemes according to WHO. Albendazole (400 mg, single oral dose) will be administered to those infected with *A. lumbricoides*, hookworm and/or *T. trichiura*.

# Conflict of interest

None of the investigators has any conflict of interest concerning the work or drug application reported in this protocol. The drugs (albendazole, mebendazole and ivermectin) as well as the placebo (aspartam) were bought from pharmacies in Switzerland and France. No drugs were donated and no drug company is involved in the formulation of this study protocol.

# Data storage and publication

All signed consent forms will remain within the office of the helminth unit of the MoHSW Zanzibar. The original data on the laboratory results will be transferred to Switzerland for entry, cleaning and analysis. A photocopy of these data will remain in the office of the helminth unit in Zanzibar. Important findings will be published in peer-reviewed open access journals. The principal investigator will be the first author and all co-investigators will be indicated as co-authors.

# References

1. N. R. de Silva *et al.*, *Trends Parasitol* **19**, 547 (Dec, 2003).

2. J. Bethony *et al.*, *Lancet* **367**, 1521 (May 6, 2006).

3. WHO, “Deworming for health and development.” (World Health Organisation, 2005).

4. P. J. Lammie, A. Fenwick, J. Utzinger, *Trends Parasitol* **22**, 313 (Jul, 2006).

5. D. Goodman *et al.*, *Am J Trop Med Hyg* **76**, 725 (Apr, 2007).

6. R. M. Anderson, May, R. M., in *Oxford University Press*. (Oxford, 1991).

7. P. J. Hotez *et al.*, *PLoS Med* **3**, e102 (Jan, 2006).

8. WHO, *Wkly Epidemiol Rec* **16**, 145 (2006).

9. G. Raso *et al.*, *Int J Epidemiol* **33**, 1092 (Oct, 2004).

10. S. Brooker, J. Bethony, P. J. Hotez, *Adv Parasitol* **58**, 197 (2004).

11. S. Brooker, A. C. A. Clements, D. A. P. Bundy, *Adv Parasitol* **62**, 221 (2006).

12. WHO, “Prevention and Control of Schistosomiasis and Soil–Transmitted Helminthiasis: Report of a WHO Expert Committee” *Tech. Report No. 29* (World Health Organisation, 2002).

13. N. J. Lwambo, J. E. Siza, S. Brooker, D. A. Bundy, H. Guyatt, *Trans R Soc Trop Med Hyg* **93**, 497 (Sep-Oct, 1999).

14. M. Booth *et al.*, *Parasitology* **116**, 85 (Jan, 1998).

15. J. Keiser *et al.*, *J Parasitol* **88**, 461 (Jun, 2002).

16. E. Renganathan *et al.*, *Bull World Health Organ* **73**, 183 (1995).

17. M. Albonico *et al.*, *East Afr Med J* **74**, 294 (May, 1997).

18. H. P. Marti *et al.*, *Am J Trop Med Hyg* **55**, 477 (Nov, 1996).

19. K. A. Mohammed *et al.*, *PLoS Negl Trop Dis* **2**, e171 (2008).

20. K. A. Mohammed, D. H. Molyneux, M. Albonico, F. Rio, *Trends Parasitol* **22**, 340 (Jul, 2006).

21. J. W. Rudge *et al.*, *Acta Trop* **105**, 45 (Jan, 2008).

22. S. Knopp *et al.*, *Geospat. Health* **3**, 47 (2008).

23. P. Christian, S. K. Khatry, K. P. West, Jr., *Lancet* **364**, 981 (Sep 11-17, 2004).

24. D. A. P. Bundy, M. S. Chan, L. Savioli, *Trans R Soc Trop Med Hyg* **89**, 521 (Sep-Oct, 1995).

25. L. Savioli *et al.*, *Trans R Soc Trop Med Hyg* **96**, 577 (Nov-Dec, 2002).

26. L. S. Stephenson, M. C. Latham, K. M. Kurz, S. N. Kinoti, H. Brigham, *Am J Trop Med Hyg* **41**, 78 (Jul, 1989).

27. L. S. Stephenson, M. C. Latham, E. J. Adams, S. N. Kinoti, A. Pertet, *J Nutr* **123**, 1036 (Jun, 1993).

28. E. A. Miguel, M. Kremer, *Econometrica* **72**, 159 (2003).

29. H. Torlesse, M. Hodges, *Trans R Soc Trop Med Hyg* **95**, 195 (Mar-Apr, 2001).

30. T. M. Atukorala, L. D. de Silva, W. H. Dechering, T. S. Dassenaeike, R. S. Perera, *Am J Clin Nutr* **60**, 286 (Aug, 1994).

31. Worldbank, *School Deworming at a Glance*, "at a glance" (Worldbank, Washington DC, 2003), pp.

32. D. A. P. Bundy, A. Hall, G. F. Medley, L. Savioli, *World Health Stat Q* **45**, 168 (1992).

33. A. Montresor, D. Crompton, A. Hall, D. Bundy, L. Savioli, *Guidelines for the Evaluation of Soil-Transmitted Helminthiasis and Schistosomiasis at Community Level* (1998), pp. 48.

34. J. Keiser, J. Utzinger, *Jama* **299**, 1937 (Apr 23, 2008).

35. A. Olsen, *Trans R Soc Trop Med Hyg* **101**, 747 (Aug, 2007).

36. J. Heukelbach *et al.*, *Bull World Health Organ* **82**, 563 (Aug, 2004).

37. M. Albonico, D. W. Crompton, L. Savioli, *Adv Parasitol* **42**, 277 (1999).

38. V. Y. Belizario *et al.*, *Bull World Health Organ* **81**, 35 (2003).

39. C. Flohr *et al.*, *Am J Trop Med Hyg* **76**, 732 (Apr, 2007).

40. M. Albonico, D. Engels, L. Savioli, *Int J Parasitol* **34**, 1205 (Oct, 2004).

41. N. Katz, A. Chaves, J. Pellegrino, *Rev Inst Med Trop Sao Paulo* **14**, 397 (Nov-Dec, 1972).

42. S. Knopp *et al.*, *PLoS Negl Trop Dis* **2**, e331 (2008).

43. S. Knopp *et al.*, *Trans R Soc Trop Med Hyg* (Jan 23, 2009).

44. J. Utzinger *et al.*, *Trans R Soc Trop Med Hyg* **102**, 84 (Jan, 2008).

45. M. Albonico *et al.*, *Trans R Soc Trop Med Hyg* **89**, 538 (Sep-Oct, 1995).

46. M. Albonico *et al.*, *Int J Epidemiol* **28**, 591 (Jun, 1999).

47. J. R. Stothard *et al.*, *Parasitology* **135**, 1447 (Oct, 2008).

48. M. Albonico *et al.*, *Bull World Health Organ* **81**, 343 (2003).

49. S. Knopp *et al.*, *Am J Trop Med Hyg* **81**, 1071 (2009).

50. M. Albonico *et al.*, *Trop Geogr Med* **46**, 142 (1994).

51. M. Albonico *et al.*, *Trans R Soc Trop Med Hyg* **88**, 585 (Sep-Oct, 1994).

52. C. Urbani, M. Albonico, *Acta Trop* **86**, 215 (May, 2003).

53. WHO, “Report of the WHO informal consultation on monitoring of drug efficacy in the control of schistosomiasis and intestinal nematodes” (WHO, 1999).

54. G. Cringoli, *Parassitologia* **48**, 381 (Sep, 2006).

55. M. J. Beach *et al.*, *Am J Trop Med Hyg* **60**, 479 (Mar, 1999).

56. J. L. Fleiss, in *Statistical methods for rates and proportions*. (Wiley, New York, 1981) pp. 212–236.

**Appendix 1: Study participant information and consent sheet (English)**

EFFICACY AND SAFETY OF DRUGS AGAINST SOIL-TRANSMITTED HELMINTH INFECTIONS IN ZANZIBAR

**Description of the research and participation:** Your child is invited to participate in a research study conducted by the Helminth Control Team Unguja (Stonetown, Zanzibar), the Swiss Tropical Institute (Basel, Switzerland), the Natural History Museum (London, UK) and the University Federico II (Naples, Italy). The purpose of this research is to re-assess the efficacy of the drug albendazole which is regularly administered against intestinal worm infections to people in Unguja. We want to find out, if the drugs albendazole and mebendazole effectively kill worms that might live in your body. Additionally, we want to assess if a combination of these drugs with ivermectin is more effective in curing intestinal worm infections and if a new diagnostic method (FLOTAC) is more reliable in detecting worm infections than the standard method (Kato-Katz). The study will ultimately help in the formulation of improved and locally-adapted control interventions.

Your child’s participation will involve the submission of 1 and in case of infection with the worm *Trichuris trichiura* a total of 4 own morning-stool samples and the completion of a questionnaire about potential adverse events of the drugs.

The amount of time required for participation will be a few minutes in the morning (7.30-8.00 before school starts) on 1 or several days (depending on infection), when stool samples are collected and children are treated against intestinal worms. Additionally, approximately 15 minutes are required for the interview to complete the questionnaire about potential side-effects of the drugs.

**Risks and discomforts:** If your child is infected with intestinal worms and treated during or at the end of the study with albendazole (400 mg, single oral dose), mebendazole (500 mg, single oral dose), albendazole plus ivermectin (200 µg/kg) or mebendazole plus ivermectin (200 µg/kg) tablets might cause stomach ache, head ache, diarrhoea, vomiting or dizziness. These symptoms will disappear after several hours.

**Potential benefits:** In case that your child is infected with intestinal worms, it will be treated to get rid of this infection. The treatment can improve your child’s general health status, including less stomach problems, less fatigue and weakness and better uptake of nutrition and thus improved school performance.

**Protection of confidentiality:** The records of your child’s infection status with intestinal worms and the questionnaire will be kept confidentially. At the end of the study your child will be informed about its infection status. Your child’s identity will not be revealed in any publication that might result from this study.

**Voluntary participation:** Your child’s participation in this research study is entirely voluntary. Your child may choose not to participate and you may withdraw your consent of your child’s participation at any time. You/your child will not be penalized in any way should you/your child decide not to participate or to withdraw from this study.

**Contact information:** If you have any questions or concerns about this study or if any problems arise, please contact the field workers. You may also wish to contact directly to Dr. Khalfan A. Mohammed - Chief at the Helminth Laboratory Office and Co-investigator of this study (Mobile Phone: + 255 777 432 370).

**I have read and understood this text and all my questions have been answered. I am aware of my rights and duties and I freely accept that my child participates in this study.**

Name of child:

Date and signature parent/legal guardian: Date and signature fieldworker:

**Appendix 2: Study participant information and consent sheet (Kiswahili)**

***MAELEZO YA UTAFITI NA KUSHIRIKI***

Mtoto wako anashirikishwa katika utafiti unaoshughulikiwa na kikundi cha Helminth Control Programme Unguja (Mji Mkongwe - Zanzibar), Swiss Tropical Institute (Basel Switzerland), Natural History Meseum (London U.K) na University Federico (Naples Italy).

Madhumuni ya utafiti ni kujua umuhimu na namna ya ufanyakazi wake (nguvu ya dawa) ya Albendazole ambayo ilozoeleka kutibu minyoo ya michango inayosababisha matatizo (maambukizo) kwa watu wa Unguja.

Lengo letu ni kujua kama dawa ya Albendazole na Mebendazole zinafanya kazi uzuri kuuwa minyoo ambayo huweza kuishi katika mwili wa binaadamu. Kwa kuengezea tunataka kujua mchanganyiko wa dawa hizi pamoja na Ivermectin zitafanya kazi uzuri zaidi kwa kutibu maambukizo ya minyoo ya michango, na kama njia mpya (FLOTAC)

Ya kutambua maradhi hayo ni nzuri zaidi kungundua minyoo ya michango kuliko njia ilozoeleka ya Kato-Katz. Utafiti huui utakuwa muhimu kuelezea uhalisi wa maendeleo ya kudhibiti maradhi hayo.

Kushiriki kwa mtoto wako atahitajika atoe choo (kinyesi) kwa mara ya kwanza na pindi akingundulika ana minyoo yanayoitwa ***Trichuris trichiura*** atatakiwa alete tena choo kwa mara nyengine. Jumla ya choo vinne vya asubuhi kwa uchukunguzi na kujibu dodosi kuhusu maendeleo ya ubora na tatizo za madawa zilizotumika.

Muda utakayotumika ni mfupi wakati wa asubuhi kama nusu saa kabla ya masaa ya skuli kwa siku ya mwanzo au siku nyenginezo inatagemea na maambukizi. Choo vitakusanywa na watoto watapata matibabu kutokana na maambukizo hayo ya minyoo ya michango, kwa kuongezea labda dakika 15 zinahitajika kwa kukamilisha dodosi kuhusu madhara ya hizo dawa.

***Hali ya hatari na maumivu madogo:***

Ikiwa mtoto wako atangundulika na minyoo ya michango na akatibiwa mwisho wa utafiti huu dawa ya Albendazole (400mg), Mebendazole (500mg), Albendazole na Ivermectin (200µg/kg) au Mebendazole na Ivermectin (200µg/kg) anaweza kupata maumivu ya tumbo, kichwa, kuharisha, kutapika na kizunguzungu. Dalili hizi zitaondoka baada ya masaa.

***Faida zitakazopatikana:***

Ikiwa mtoto wako kapata maambukizi ya minyoo ya michango atatibiwa na atapona, matibabu yatamsaidia mtoto wako kupata afya bora ikiwemo kupunguza maumivu ya tumbo, uchofu. udhaifu na kupata afya bora na kumletea maendeleo mazuri skuli.

***Kuhifadhi kwa siri:***

Matokeo yote kuhusu maambukizi ya minyoo ya michango kuhusu mtoto yataekwa siri. Na mwisho wa utafiti huu mtoto wako atapatiwa majibu yake kuhusu maambukizo.

***Kushiriki:***

Kushiriki kwake mtoto wako ni kwa hiari na pindi akikataa kushiriki katika utafiti huu hatolipishwa wala hatopewa adabu ya aina yoyote.

***Maelezo ya ziada:***

Ikiwa una tatizo lolote suali linalohusiana na utafiti huu tafadhali onana na Mkuu wa Kitengo cha Kichocho, Minyoo na Matende Ndugu Khalfan.A.Moh’d au piga simu Namba +255777432370.

Nimesoma na nimefahamu yote na masuali yangu yamejibiwa nipo tayari kukubali na kushiriki katika utafiti huu.

**Jina la mtoto: ..........................................................................................................................**

**Tarehe na Sahihi ya Mzee: Tarehe na Sahihi ya Mfanyakazi:**

**................................................. ....................................................**

**Appendix 3: Detailed field and laboratory procedures**

**Week 1**

Before start of the fieldwork:

- Information of local authorities, headmasters of school and teachers

- Ask for a list of pupils from all grades

- Select randomly about 2000 children

- Prepare FLOTAC solution S4 (sodium nitrate: NaNO_3_ 315 g plus 685 ml H_2_O; specific density = 1.20)

- Prepare SAF solution (15g Sodium acetate, 20 ml acetic acid (glacial), 53.3 ml formaldehyde solution (35%), 911.7 ml H_2_O) and pre-fill tubes (10 ml per tube)

**Week 2**

Fieldwork:

- Explain the study to school children and distribute consent forms

- Obtain written informed consent from parents/legal guardians of each of the selected pupils

- Distribute “ID cards” and stool collection containers numbered with ID to the 1^st^ 200 pupils and ask for a large stool sample (lemon size)

Identification (ID) numbers:

- 6 digits and 1 letter:

- First digit: Grade number (1-6)

- Second digit: K for Kinyasini school, C for Chaani school

- Digit three to six: individual ID (assigned after informed consent, start with 0001, 0002, 0003 etc. up to 2000 until all individuals have received a unique number)

**Week 2 to 5**

- Collect filled stool containers from 1^st^ 200 pupils and record them on the form

- Hand out labelled stool collection containers to 2^nd^ 200 participants

- Transport collected full stool containers to the laboratory

- Prepare 2 Kato-Katz slides from each stool sample as described beyond

- Preserve 1 g of stool in SAF from ~250 randomly selected children

🡪 Repeat the procedure until 500-600 children infected with *T. trichiura* are identified (approximately when stool samples of 2000 children are examined)

**Week 5 and 6**

- Prepare a list containing the IDs of the 500-600 children infected with *T. trichiura*

- Prepare a list containing the IDs of the remaining children infected with *A. lumbricoides* and/or hookworm

- Assign the 500-600 children infected with *T. trichiura* randomly to one of the four treatment regimens

- Assign the remaining children infected with *A. lumbricoides* and/or hookworm randomly to one of the four treatment regimens

- Check children infected with *T. trichiura*, *A. lumbricoides* and/or hookworm for exclusion criteria

- Distribute labelled stool container to all children included into the randomized trial

- Collect filled stool containers from all children and transport them to the laboratory

- Prepare 2 Kato-Katz slides from each stool sample and examine as described beyond

- Distribute the drugs to the children according to the randomized list

- Ask child to swallow the drug with clean water and check that the tablet has been swallowed by looking into the mouth and under the tongue

- Monitor and record side effects for 3 hours provide medical assistance where necessary

**Week 7 and 8**

- Monitor and record side effects 3 days past treatment

- Examine the 250 SAF preserved stool samples with the FLOTAC method

**Week 9 and 10**

- Distribute labelled stool container to all children treated in the randomized trial

- Collect filled stool containers from all children and transport them to the laboratory, distribute a labelled new stool container for every one that was collected until each child submitted 2 stool samples after treatment

- Prepare 2 Kato-Katz smears from each stool sample and examine as described beyond until 2 consecutive stool samples from all children included into the trial were examined with the Kato-Katz method

- Preserve 1 g of 1 stool sample in a labelled container with 10ml SAF from the sub-sample of children randomly selected to be examined with FLOTAC and which finally entered the trial

- Inform participants in person about the parasitological diagnosis

- Treatment of all participating school-children free of charge with albendazole by members of the national helminth control team

**Week 11 and 12**

- Examine the SAF preserved stool samples from the days of follow up with the FLOTAC method

Laboratory procedures:

Kato-Katz slide

- Prepare 2 Kato-Katz slides from every stool sample (baseline screening, day of treatment, days of follow up)

- Read for hookworm within 20-30 minutes (hookworm eggs clear fast). Other eggs can be read later. Magnification: 100x

- Keep slides for quality control

- Count all eggs, no estimation after partial reading

- Record result on form

- 10% of slides are re-read by senior technician for quality control

SAF conservation

- Label tube containing 10 ml SAF solution with ID

- Fill 1-2 g stool into the tube

- Mix well

FLOTAC

- Pass SAF preserved stool through an aperture with 350 µm in order to remove large fibres

- Add 10 ml of SAF to 1 g of stool

- Pipette this stool-SAF suspension into a 15 ml Falcon tube

- Centrifuge for 3 min at 170 g

- Discard supernatant

- Fill tube to the 6 ml mark with S4

- Mix pellet accurately and transfer 5 ml into one of the two chambers of the FLOTAC apparatus

- Record which sample was filled into which chamber of which apparatus

- Centrifuge apparatus for 5 min at 120 g

- Translate the top portion of the flotation chambers and remove rest of apparatus

- Read observation grids at a 100x magnification

- Record and count helminth eggs separately for each species

**Appendix 4: Questionnaire on side effects**


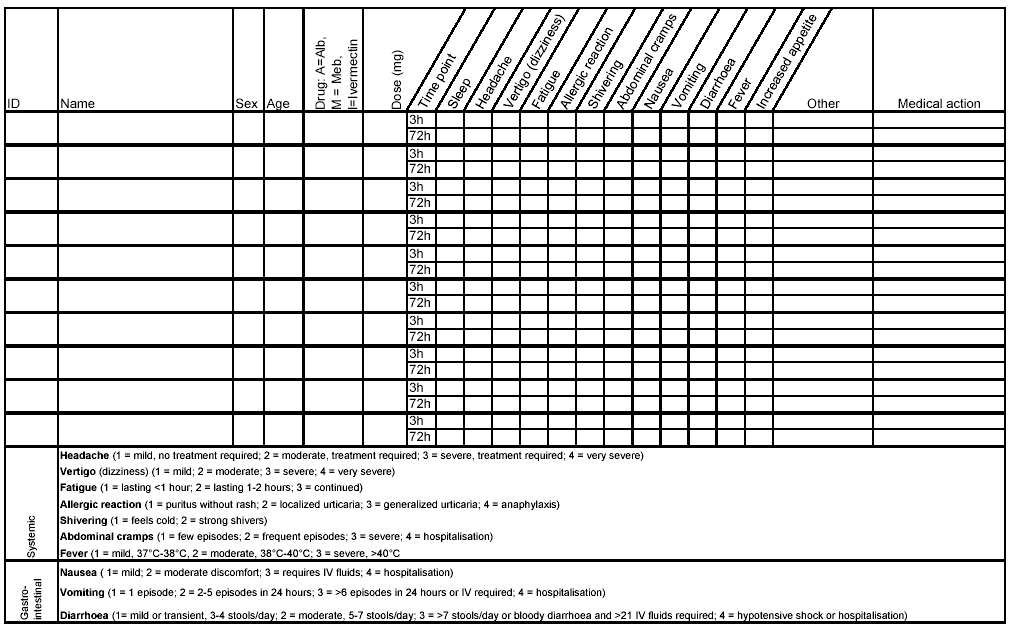


**Appendix 5: Overview participation**

| **ID** | **Name** | **Sex** | **Age** | **Sample A (No. K-K)** | **RCT**  **(Yes/No)** | **Sample B (No. K-K)** | **Sample A (FLOTAC)** | **Treatment**  **(Alb, Meb, Alb+Ivm, Meb+Ivm)** | **Sample C (No. K-K)** | **Sample D (No. K-K)** | **Sample C/D (FLOTAC)** |
| --- | --- | --- | --- | --- | --- | --- | --- | --- | --- | --- | --- |
| **0001** |  |  |  |  |  |  |  |  |  |  |  |
| **0002** |  |  |  |  |  |  |  |  |  |  |  |
| **0003** |  |  |  |  |  |  |  |  |  |  |  |
| **0004** |  |  |  |  |  |  |  |  |  |  |  |
| **0005** |  |  |  |  |  |  |  |  |  |  |  |
| **0006** |  |  |  |  |  |  |  |  |  |  |  |
| **0007** |  |  |  |  |  |  |  |  |  |  |  |
| **0008** |  |  |  |  |  |  |  |  |  |  |  |
| **0009** |  |  |  |  |  |  |  |  |  |  |  |
| **0010** |  |  |  |  |  |  |  |  |  |  |  |
| **0011** |  |  |  |  |  |  |  |  |  |  |  |

**Appendix 6: Overview laboratory results**

**Parasitological survey Zanzibar 2009**  **Kato-Katz**

**Technician name: Date:**

| **ID** | ***Ascaris*** | ***Trichuris*** | **Hookworm** | ***Enterobius*** | ***Taenia*** |
| --- | --- | --- | --- | --- | --- |
|  |  |  |  |  |  |
|  |  |  |  |  |  |
|  |  |  |  |  |  |
|  |  |  |  |  |  |
|  |  |  |  |  |  |
|  |  |  |  |  |  |
|  |  |  |  |  |  |
|  |  |  |  |  |  |
|  |  |  |  |  |  |
|  |  |  |  |  |  |
|  |  |  |  |  |  |
|  |  |  |  |  |  |
|  |  |  |  |  |  |
|  |  |  |  |  |  |
|  |  |  |  |  |  |
|  |  |  |  |  |  |
|  |  |  |  |  |  |
|  |  |  |  |  |  |
|  |  |  |  |  |  |
|  |  |  |  |  |  |
|  |  |  |  |  |  |
|  |  |  |  |  |  |
|  |  |  |  |  |  |

**Appendix 7: Overview laboratory results**

**Parasitological survey Zanzibar 2009**  **FLOTAC**

**Technician name: Date:**

| **ID** | ***Ascaris*** | ***Trichuris*** | **Hookworm** | ***Strongyloides*** | ***Enterobius*** | ***Taenia*** |
| --- | --- | --- | --- | --- | --- | --- |
|  |  |  |  |  |  |  |
|  |  |  |  |  |  |  |
|  |  |  |  |  |  |  |
|  |  |  |  |  |  |  |
|  |  |  |  |  |  |  |
|  |  |  |  |  |  |  |
|  |  |  |  |  |  |  |
|  |  |  |  |  |  |  |
|  |  |  |  |  |  |  |
|  |  |  |  |  |  |  |
|  |  |  |  |  |  |  |
|  |  |  |  |  |  |  |
|  |  |  |  |  |  |  |
|  |  |  |  |  |  |  |
|  |  |  |  |  |  |  |
|  |  |  |  |  |  |  |
|  |  |  |  |  |  |  |
|  |  |  |  |  |  |  |
|  |  |  |  |  |  |  |
|  |  |  |  |  |  |  |
|  |  |  |  |  |  |  |
|  |  |  |  |  |  |  |
|  |  |  |  |  |  |  |

**Appendix 8: Flowchart of participants infected with *T. trichiura* and included in the RCT**

Baseline screening of pupils from Kinyasini school

(n = 1800)

Children infected with *T. trichiura*

Random allocation to **4** treatment groups

(n = 540)

Children **not** infected with *T. trichiura*

(expected **prevalence 30%;** n = 1260)

Children falling under **exclusion** criteria

(5%; n = 7)

Albendazole

(n = 135)

Albendazole and Ivermectin

(n = 135)

Mebendazole

(n = 135)

Cure rate?

Egg reduction rate?

(n = 103)

Cure rate?

Egg reduction rate?

(n = 103)

Cure rate?

Egg reduction rate?

(n = 103)

Participants **not** complying

(10%; n = 14)

Albendazole treatment

(n = 114)

ALB + IVM treatment

(n = 114)

Mebendazole treatment

(n = 114)

Children falling under **exclusion** criteria

(5%; n = 7)

Participants **not** complying

(10%; n = 14)

Children falling under **exclusion** criteria

(5%; n = 7)

Participants **not** complying

(10%; n = 14)

Random selection of pupils from Chaani and Kinyasini school

(n = 2000)

Children **not** complying

(expected non-compliance of 10%; n = 100)

Participants **not** complying

(10%; n = 11)

Participants **not** complying

(10%; n = 11)

Participants **not** complying

(10%; n = 11)

Mebendazole and Ivermectin

(n = 135)

Participants **not** complying

(10%; n = 14)

Children falling under **exclusion** criteria

(5%; n = 7)

MEB + IVM treatment

(n = 114)

Participants **not** complying

(10%; n = 11)

Cure rate?

Egg reduction rate?

(n = 103)
